# Mapping the gendered dynamics of cesarean section: A scoping review

**DOI:** 10.1371/journal.pgph.0006634

**Published:** 2026-07-17

**Authors:** Noah M. Trudeau, Kirantheja Daggula, Indira Prihartono, Rosemary Morgan, Shatha Elnakib

**Affiliations:** Department of International Health, Johns Hopkins Bloomberg School of Public Health, Baltimore, Maryland, United States of America; UNAM: Universidad Nacional Autonoma de Mexico, MEXICO

## Abstract

Cesarean section (CS) is a lifesaving obstetric intervention when medically indicated, yet global rates have risen sharply over recent decades, with substantial variation across and within countries. While underuse persists in many low-resource settings, overuse is increasingly documented in higher-income and private-sector facilities, raising concerns about inequitable access, unnecessary surgical intervention, and divergent quality of maternity care. Existing explanations have largely focused on clinical indications and biomedical risk profiles, often neglecting the social and gendered structures that shape decision-making, institutional practice, and access to care. This scoping review examines how gender norms, roles, and power relations influence CS utilization across diverse global contexts. We conducted a scoping review following PRISMA-ScR guidelines, searching PubMed, Scopus, and CINAHL for English- and French-language publications from 2000 to 2025. Studies were included if they explicitly examined gender-related drivers or constraints shaping CS access, decision-making, or delivery practices. A three-concept search strategy captured cesarean delivery, gendered constructs, and mechanisms through which gender operates in health systems and social contexts. Data were extracted using a structured matrix and synthesized thematically through a gender analysis framework spanning access to resources, roles and practices, norms and beliefs, decision-making power, and institutions, while distinguishing between gendered “push” and “pull” factors influencing CS use. 95 studies met inclusion criteria. The evidence demonstrates that CS utilization is shaped by interacting gendered forces operating across individual, household, community, and health system levels. Four interrelated domains emerged. First, political and economic structures—including financing models, privatization, and provider incentives—shape institutional preferences for surgical delivery and normalize medicalized childbirth. Second, clinical cultures marked by medical paternalism, risk aversion, and medico-legal pressures shift decision-making authority from women to providers. Third, household and community gender relations structure reproductive decision-making through spousal authority, familial pressure, and socially embedded norms of motherhood, sexuality, and bodily integrity. Fourth, women’s preferences are shaped by both enabling and constraining conditions, including time poverty, fear of labor pain, concerns about sexual and reproductive health, and uneven access to respectful maternity care. Across contexts, CS emerges as both overused and underused depending on women’s social position, access to resources, and exposure to institutional power. Wealth, urban residence, and private insurance often facilitate elective CS, while poverty, geographic isolation, and weak health systems restrict access even when clinically necessary. Gendered norms simultaneously construct vaginal birth as morally valued and CS as either a marker of modernity or medical failure, reinforcing contradictory pressures on women and providers alike. This review highlights that CS is not solely a clinical outcome, but a socially produced intervention embedded within gendered systems of power. Addressing inequities in CS requires interventions that extend beyond clinical guidelines to include health financing structures, institutional accountability, provider norms, and the broader social conditions that shape reproductive agency.

## Introduction

Cesarean section (CS) is a critical, lifesaving intervention when clinically indicated, capable of preventing maternal and neonatal morbidity and mortality. Over recent decades, global CS rates have risen substantially, reflecting expanded access to facility-based childbirth and the growing availability of obstetric interventions [1]. Currently, an estimated 21% of all births worldwide are delivered by CS, with significant regional variation: from as low as 5% in sub-Saharan Africa to 42% in Latin America and the Caribbean [2]. Projections suggest that by 2030, nearly 28% of births – approximately 38 million deliveries annually – will be conducted via CS, with the majority occurring in low- and middle-income countries [2]. These patterns challenge long-standing WHO benchmarks, which posit that CS rates above 10–15% at the population level are unlikely to confer additional mortality benefits, while rates below this threshold may indicate insufficient access to essential obstetric care [2].

Rising CS rates have raised concern that certain procedures are performed without medical indication, reflecting the increasing medicalization of childbirth rather than clinical need [3,4]. When unnecessary, CS exposes women and newborns to avoidable risks, including infection, hemorrhage, anesthesia-related complications, and longer-term reproductive sequelae [3,4]. Conversely, persistently low CS rates in some health systems signal ongoing barriers to facility-based delivery and comprehensive emergency obstetric services [5]. The simultaneous overuse and underuse of CS underscores profound inequities in access to quality maternity care and women’s reproductive outcomes.

Although much research has examined CS utilization through clinical indications and obstetric risk profiles, such approaches often overlook the social and gendered logics influencing childbirth [6–8]. In this review, we draw on the WHO’s distinction between *sex* – the biological attributes associated with chromosomes, hormones, and reproductive anatomy – and *gender*, defined as the socially constructed norms, roles, and power relations that structure interactions between women, men, boys, girls, and gender-diverse people [9]. Qualitative studies reveal that women’s preferences for surgical birth are not solely biomedical decisions but are negotiated within broader social and institutional contexts [10,11]. In settings where women experience time poverty due to unpaid care work, employment obligations, and reproductive labor, CS may serve as a mechanism to exert control over the timing and conditions of childbirth, aligning reproductive choices with domestic and professional responsibilities [11]. In this sense, birth preferences cannot be understood as purely elective; they are shaped by the constraints and expectations imposed by gendered social structures.

Birth modes also carry social and moral significance that varies across cultural contexts. CS can signify modernity, status, or access to higher-quality care, whereas vaginal birth may be valorized as an expression of feminine strength, moral virtue, or maternal dedication [12,13]. These symbolic meanings interact with supply-side push and pull factors: health system–level incentives, constraints, and professional norms that either encourage (“push”) or discourage (“pull”) the use of surgical delivery independent of strict clinical indication. Importantly, provider decision-making is also shaped by gendered assumptions. Qualitative research suggests that clinicians may frame women as overly fearful of labor pain, insufficiently informed to participate in complex decisions, or in need of authoritative guidance [14,15]. These patterned expectations about women’s bodies, endurance, credibility, and social worth influence counseling, consent processes, and ultimately the likelihood of CS [16].

Despite the complexity and global significance of these patterns, the gendered dimensions of CS decision-making and access remain under-explored. A gender lens offers an essential analytic framework for explaining why CS is simultaneously overused among some populations and underused among others. Without accounting for how power relations, social roles, and systems of valuation structure both demand- and supply-side decision-making, prevailing explanations risk reducing CS trends to individual preference or clinical necessity alone. This scoping review addresses this gap by systematically examining how gender operates across the push and pull factors influencing CS delivery. The central question guiding this inquiry is: How do gender-related norms, roles, and power relations shape access to and utilization of CS across diverse contexts?

## Methods

We conducted a scoping review of the literature to map the evidence on how gender norms and inequities intersect with health systems, medical practice, and social hierarchies to shape patterns of surgical birth. Conducted in August 2025, the review was guided by the *PRISMA-ScR* (Preferred Reporting Items for Systematic Reviews and Meta-Analyses extension for Scoping Reviews) guidelines [17].

We developed a three-concept search strategy to explicitly capture gendered drivers and deterrents of CS. Controlled vocabulary (MeSH) were used to ensure comprehensive retrieval of studies addressing CS. Concept 1 (C-Section) captured the full range of clinical terminology related to cesarean delivery, including elective and emergency procedures, trial of labor after cesarean, and related operative birth terms. Concept 2 (Gender) focused on sex- and gender-related constructs, including gender norms, roles, power relations, autonomy, patriarchy, and gender inequality. Concept 3 (Gendered Influences) targeted mechanisms through which gender operates to shape CS utilization, which are summarized in Table 1.

**Table 1:** Database search strategy and search terms.

Concept	Syntax
Concept 1: Cesarean Section	cesarean section OR caesarean section OR c-section OR cesarean OR caesarean OR cesarean delivery OR caesarean delivery OR surgical birth OR operative delivery OR elective cesarean OR emergency cesarean OR cesarean section rate OR trial of labor OR trial of labor after cesarean OR TOLAC OR vaginal birth after cesarean OR VBAC
Concept 2: Gender	gender OR sex factors OR sex differences OR gender norms OR gender roles OR gender bias OR gender equity OR gender inequality OR gender power relations OR femininity OR patriarchy OR women’s autonomy OR reproductive autonomy OR bodily autonomy OR women’s roles OR gender dynamics
Concept 3: Gendered Influences	power OR stigma OR social determinants of health OR informed consent OR health personnel attitudes OR health knowledge attitudes practice OR reproductive rights OR patient rights OR empowerment OR disempowerment OR coercion OR time poverty OR reproductive labor OR domestic work OR sexual norms OR sexual desirability OR status symbols OR modernity OR abuse OR mistreatment OR obstetric violence OR provider bias OR structural barriers OR financial incentives OR cost barriers OR pathologization OR overmedicalization OR birth preferences OR maternal decision-making OR provider attitudes OR institutional norms OR medical paternalism OR medicalization of childbirth OR reproductive justice OR maternal rights OR gender-responsive care

All three concepts were combined using Boolean operators and applied consistently across three databases. Searches were limited to English- and French-language publications from 2000–2025 via PubMed, Scopus, and the Cumulative Index to Nursing and Allied Health Literature (CINAHL). Full search strings for each database are provided in S1 Table. All records were imported into Covidence systematic review software for de-duplication, screening, and data extraction. Titles and abstracts were screened independently by two reviewers against predefined inclusion and exclusion criteria, followed by full-text review. Discrepancies were resolved by two additional reviewers.

Studies were eligible for inclusion if they explicitly examined gender-related drivers, deterrents, or dynamics influencing CS utilization, including elective and medically indicated procedures. We included studies that analyzed how gender norms, roles, power relations, or autonomy shaped women’s decision-making, provider behavior, or institutional practices at individual, community, or health system levels. Articles addressing intersections between gender and other social axes – such as socioeconomic status, age, race or ethnicity, marital status, or geographic location – were also included. Eligible study designs included qualitative, quantitative, and mixed-methods empirical research; theoretically grounded conceptual papers; and policy or systems analyses with an explicit gender lens. There were no geographic restrictions.

**Fig 1:**
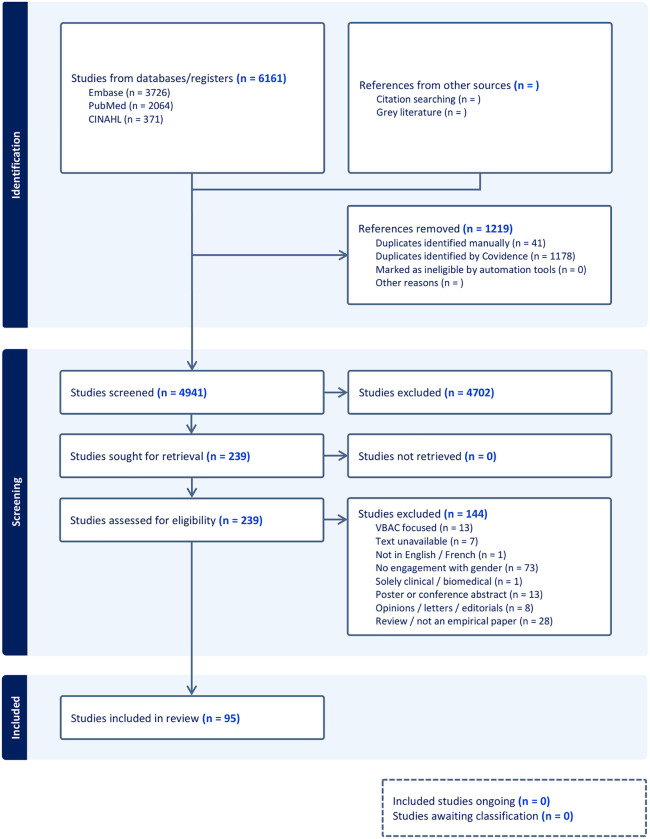
PRISMA diagram.

We excluded studies focused on clinical indications, biomedical risk factors, or technological interventions without attention to gendered processes. Articles addressing post-operative outcomes or neonatal care were excluded unless findings were explicitly linked to gendered decision-making or access to CS. Editorials, opinion pieces, and conference abstracts were also excluded (Fig 1).

Data extraction was conducted using a structured extraction matrix developed and piloted within Covidence. Extracted variables included author, year, title, country and region, study and document type, research objectives, population, methodology, and CS type (elective, emergency, or both). To support gender-focused synthesis, the matrix explicitly captured gender-based drivers and deterrents of CS at three levels of analysis: individual, community, and health system. Additional fields captured key findings, recommendations, and analytic notes.

Extracted data were then synthesized using an established gender analysis matrix approach [18–20]. Findings were organized across five domains of gender analysis: access to resources; roles and practices; norms, values, and beliefs; decision-making power and autonomy; and laws, policies, and institutions [19].

To capture how these dynamics shape both overuse and underuse of CS, themes were further organized into two overarching categories: gendered push factors, which encourage or increase the likelihood of CS uptake (whether medically indicated or not), and gendered pull factors, which discourage or constrain access to CS even when clinically necessary. For example, financial incentives for providers and hospitals to perform surgical deliveries were coded under the *access to resources* domain when they intersected with norms that devalue women’s time and autonomy. Similarly, institutional practices such as predictable surgical scheduling, staffing shortages that limit continuous labor support, or clinical routines that prioritize operative efficiency were mapped to the *roles and practices* domain, reflecting how provider workflows and professional routines shape delivery mode.

Cultural meanings attached to childbirth were mapped under the *norms, values, and beliefs* domain. For instance, studies describing cesarean birth as a symbol of modernity, safety, or social prestige – or conversely portraying vaginal birth as a moral demonstration of feminine endurance – were categorized within this domain as gendered narratives influencing women’s preferences and social expectations surrounding birth. Household and interpersonal dynamics, including spousal authority over reproductive decisions or family pressure discouraging surgical birth due to stigma or cost, were coded under *decision-making power and autonomy*, highlighting how gendered power relations within families affect women’s ability to consent to or request CS. Finally, broader structural conditions – such as weak regulation of private maternity markets, liability environments that encourage defensive obstetrics, or under-resourced public-sector emergency obstetric care – were mapped to the *laws, policies, and institutions* domain, reflecting how governance and health system organization shape patterns of CS use.

**Fig 2:**
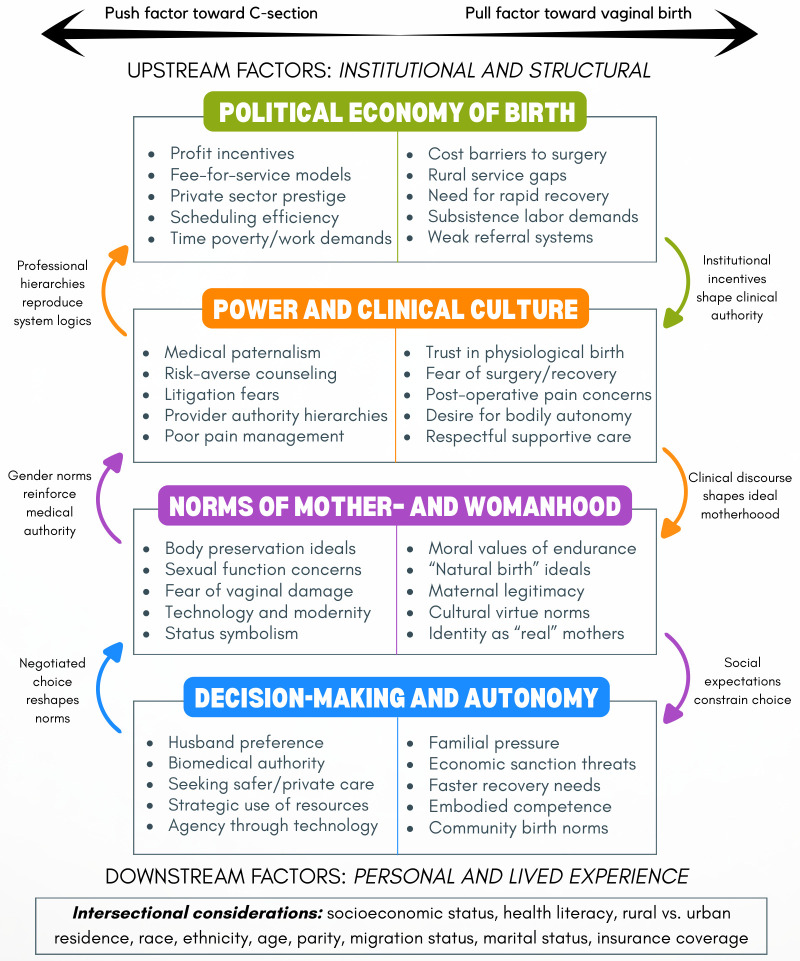
Gendered push and pull factors of cesarean section and vaginal birth.

Intersectional characteristics – including socioeconomic status, education, geographic location, age, and ethnicity – were also documented during coding to identify how gender interacts with other axes of inequality to produce differential patterns of CS access and utilization. This matrix-based approach enabled a structured synthesis of diverse qualitative and quantitative evidence while maintaining analytic attention to the multiple social, institutional, and economic pathways through which gender influences CS decision-making. This is illustrated in Fig 2 and S2 Table.

## Results

The database search yielded a total of 6,161 records, including 3,726 from Embase, 2,064 from PubMed, and 371 from CINAHL. Following de-duplication, 1,219 records were removed, including 1,178 duplicates identified by Covidence and 41 duplicates identified manually. 4,941 records remained and were screened by title and abstract, of which 239 full-text studies were assessed. A total of 144 were excluded for the following reasons: lack of engagement with gender-related concepts (n = 73); reviews or non-empirical papers (n = 28); posters or conference abstracts (n = 13); studies focused exclusively on vaginal birth after cesarean (VBAC) (n = 13); opinion pieces, editorials, or letters (n = 8); unavailable full text (n = 7); publications not in English or French (n = 1); and studies focused solely on clinical or biomedical factors without social or gender analysis (n = 1). A total of 95 studies met the inclusion criteria and were included in the final scoping review, as detailed in S3 Table.

### Descriptive results

The key descriptive characteristics of the 95 included studies – including level of analysis, study design, year of publication, country income classification, and study populations – are summarized in Table 2. Overall, the literature spans a 25-year period and reflects a marked expansion over time.

**Table 2:** Study characteristics and analytical scope of included literature (n = 95).

Level of Analysis	Count^a^
Individual	91
Health System	73
Community	62
**Types of Methods**	**Count**
Primary Data (Mixed-Methods)	14
Primary Data (Qualitative)	36
Primary Data (Quantitative)	37
Secondary Analysis	8
**Year of Publication**	**Count**
2000-2009	22
2010-2019	46
2020-Present	27
**Economic Profile of Country Studied**	**Count**
Low-Income	3
Lower-middle Income	22
Upper-middle Income	27
High Income	43
**Study Population(s)**	**Count**
Pregnant People	81
Partners/Family of Pregnant People	9
Healthcare Providers	25
Other	8

^a^Some category totals do not sum to 100% (n = 95) because several studies spanned more than one analytical level or population group.

Studies were conducted in more than 30 individual countries and included five multi-country analyses. The highest number of studies were conducted in Nigeria (n = 10), Iran (n = 8), Sweden (n = 7), Australia (n = 7), and the United States (n = 7). Other frequently represented countries included Canada (n = 4), China (n = 4), Brazil (n = 4), Argentina (n = 3), Taiwan (n = 3), Turkey (n = 3), and Norway (n = 3). The remaining studies were distributed across a wide range of settings in Latin America, sub-Saharan Africa, South and Southeast Asia, the Middle East, and Europe, with many countries represented by one or two studies.

Pregnant people were the most frequently studied population (n = 81). Healthcare providers were included in 25 studies, either alone or in combination with pregnant people, while partners or family members of pregnant people were included in 9 studies. A smaller number of studies focused on other populations, including community leaders, activists, university students of reproductive age, media users, and reproductive-age individuals not currently pregnant.

### Gendered Political Economy of Birth

A gendered political economy framework situates childbirth within market logics, institutional incentives, resource constraints, and gendered divisions of labor, creating differential opportunities and constraints for women’s reproductive agency. Applying this lens to CS allows us to interrogate how structural inequalities – such as income disparities, labor markets, provider remuneration models, and clinical hierarchies – interact with gendered norms to shape both demand for and access to surgical birth.

### Commodification and Medicalization of Labor

The rise in C-sections across private and mixed systems is closely tied to financial incentives and medicalized understandings of risk that structurally privilege surgical delivery, often at the expense of maternal agency and physiological birth [21–28]. Across middle- and upper-income settings, CS rates are consistently higher in private than public facilities, where provider remuneration, hospital profit structures, and fee-for-service payment models make surgical delivery more financially rewarding [24,25,29–33]. In Mexico, for example, the substantially higher remuneration for C-sections – three times that of vaginal delivery – has normalized surgical intervention. As one participant in Oaxaca observed, “*a normal delivery costs 5000 pesos and a C-section costs 15,000. It has turned into a business”* [34].

Beyond economic motivations, the medicalization of childbirth also drives CS rates by framing surgical birth as an efficient, predictable, and technologically advanced mode of delivery [26,28–30,35–46]. Within such clinical discourses, professional authority and risk-based messaging often shaped how women make birth mode decisions. Across eight Australian public hospitals, Thirukumar et al. (2021) found that while most women preferred vaginal birth, many frequently submitted to clinicians’ recommendations when C-sections were framed as safest for the baby, illustrating how medicalized risk discourses can override maternal agency and embed gender norms into clinical practice [47].

### Stratified access to surgical birth

Patterns of CS access reveal simultaneous push and pull dynamics structured by socioeconomic stratification. Financial resources, private insurance, and urban residence facilitate access to surgical birth, effectively pushing wealthier women toward CS by presenting it as both available and desirable [27,28,48–50]. Across settings, CS was described as a marker of status or superior care, with the ability to pay enabling women to bypass institutional bottlenecks and secure elective procedures [24,25,27–29,33–35,37–40,42,48–60].

Conversely, structural exclusion pulls women in low-resource and rural settings toward vaginal birth due to prohibitive out-of-pocket costs and non-functioning referral networks [22,26,34,40,48,49,53–55,59,61–71]. Age further complicates these dynamics; despite expressing higher preferences for CS, adolescents and young adults were more likely to experience delayed or denied access to CS in Brazil, Argentina, Canada, Chile, and Iran [29,41,49,72,73]. In a cross-sectional survey of 204 pregnant women attending routine prenatal care in Iran, younger age at marriage was significantly associated with a preference for cesarean delivery [49]. The authors suggest that this preference reflects cultural beliefs that exaggerate the risk of a narrow pelvis in younger brides, potentially overstating the likelihood of cephalopelvic disproportion and the need for surgical intervention [49].

### Care Economy and Convenience

Gendered divisions of labor generate push and pull pressures around birth mode [21,28,33,35,48,71,74,75]. Across in-depth interviews with 20 first-time Taiwanese mothers, Huang et al. (2013) found that elective cesarean delivery was used to manage time poverty, enabling women to synchronize birth with work and childcare responsibilities [35]. Yet, in subsistence-based economies like rural Bangladesh, these same domestic labor expectations acted as a powerful deterrent to surgical intervention [33]. Across focus groups conducted by Begum et al. (2018), pregnant women emphasized that the heavy physical demands of subsistence work – such as agricultural processing and livestock management – made cesarean recovery highly burdensome, with one noting, *“women’s lives become really handicapped after having this operation”* [33].

At the health system level, clinical convenience was a repeated driver of provider behavior. In high-volume or understaffed environments, the predictability of scheduled surgery aligns with hospital workflows and reduces the time demands of monitoring prolonged labor [23,26,31,37,39,50,51,59,68,73,74,76–83]. In Bagheri et al.’s 2013 qualitative study of Iranian obstetricians, one participant explained that CS “*gives us the opportunity to manage our schedules, finding someone to work instead of us, [and] tell the hospital when we are leaving*,” reflecting a perceived practical advantage of CS in the context of clinical workload and time management [51]. These professional incentives converge with women’s domestic constraints to produce mutually reinforcing pressures that normalize operative birth.

### Paternalism and Clinical Culture

Across clinical settings, entrenched patriarchal structures and hierarchical power dynamics shape cesarean delivery practices. These gendered dynamics are reinforced through medical paternalism, risk-averse practices, and socio-legal pressures, which collectively consolidate authority over CS within professional hierarchies and limit women’s agency.

### Physician Gender and Medical Paternalism

Medical paternalism refers to a model of clinical authority in which healthcare providers make decisions on behalf of patients, justified by presumed superior knowledge and expertise of the clinician [68]. Across the included literature, some clinicians assume responsibility for assessing risk, determining the ‘optimal’ mode of delivery, and directing women’s behavior, frequently under the guise of safeguarding maternal or neonatal outcomes [28,32,33,37,38,48,61,64,68,72,75,77,84–91].

Three studies cited physician gender as a significant structural variable influencing CS uptake [55,92,93]. Using chart review data from 1,000 deliveries at Yale-New Haven Hospital, Mitler et al. (2000) found that male obstetricians were significantly more likely than their female colleagues to perform cesarean sections [92]. Similarly in Taiwan, across 857,920 singleton births – that is, pregnancies involving a single fetus – without clinical indications for CS, Liu et al. (2008) found that male obstetricians demonstrated a higher propensity to perform CS upon maternal request than female counterparts [55]. The strongest associations were observed in lower-level facilities such as district hospitals (OR = 1.53) and clinics (OR = 2.26) [55].

These findings reflect broader paternalistic dynamics in obstetric care. Such dynamics were stratified by patient characteristics, including age, marital status, and parity [28,29,35,36,38,40,41,49,72,73,81,82,93–101]. In a qualitative study of immigrant women in Melbourne, Australia, younger, unmarried, and primiparous (first pregnancy) women were often positioned at the lower rungs of the clinical hierarchy, with their birth preferences subordinated to ‘expert-led’ surgical decision-making [42]. One Lao participant reflected on her cesarean experience, stating, “*I just leave it with the doctor to decide for me...They just gave me a consent form to sign, and I signed it*” [42].

### Medico-Legal Environment and Defensive Obstetrics

The shift toward CS in many settings is reinforced by a globalized medico-legal climate that encourages surgical intervention out of fear of medical liability. Research suggests that providers increasingly engage in defensive obstetrics, utilizing CS as a preemptive risk-mitigation strategy to shield against litigation or institutional sanction [32,33,37,51,68,80].

This push toward surgical birth was found to be especially pronounced in private-sector facilities and high-income urban centers in the United Kingdom, Switzerland, Argentina, and Hungary, where legal frameworks permit a higher frequency of malpractice claims [32,37,51,68,80]. The pressure of this environment is encapsulated by one physician, who notes that “*If a patient sues us for any reason, the first thing they ask us is why you didn’t have [a] caesarean section*” [51]. Consequently, clinicians – motivated by fears of professional and financial repercussion – often routinize surgical delivery as the default birth mode, prioritizing legal protection over women’s recovery or preferences.

### Managing Birth Pain and Fear

The anticipation of labor pain emerged as one of the most pervasive drivers of CS [23,27,43,52,53,56,60,61,65,70,72,76,79,87,93,95,98,101–107]. Two qualitative studies from Nigeria and Iran documented that the systemic lack of accessible labor analgesics contributes to women’s traumatic childbirth experiences [51,53]. Inadequate pain relief can lead to tokophobia (pathological fear of childbirth), rendering CS as the only perceived alternative to physical distress [41,65,82,103]. However, fear of labor pain interacts with previous birth experiences and cultural notions of womanhood in complex ways. For example, Enabudoso et al. (2011) administered a questionnaire to 139 Nigerian women at their first prenatal visit and found that among those with a prior cesarean delivery, approximately one-quarter would decline a medically indicated repeat procedure, citing postoperative pain as the primary concern and the perceived “failure of womanhood” as a secondary reason [53].

Tokophobia drives demand for elective CS among wealthier women, who leverage financial resources to bypass the perceived trauma of public labor wards [32,35,36,65,98,103]. Across low-resource or marginalized ethnic communities in Iran, Nigeria, Australia, and Mexico, women’s labor pain is frequently pathologized or dismissed by healthcare providers, undermining trust in the health system [42,69,98,102,108]. As one mother explained, “*Speaking harshly to women will make them suppress their questions. They will be dying in silence*,” illustrating how poor relational care undermines trust and may deter women from natural birth [69]. Importantly, fears around pain, disrespectful communication, and limited engagement with providers reflect systemic deficits in respectful maternity care, which emerges as a compelling driver of CS.

### Norms of Womanhood, Motherhood, and Sexuality

CS is deeply embedded within social and cultural constructions of womanhood, motherhood, and sexuality. Women’s experiences and decisions surrounding birth mode are shaped by societal expectations regarding maternal virtue, bodily aesthetics, and sexual identity, highlighting how cesarean and vaginal deliveries are differently valorized across contexts.

### Social Meanings of Cesarean and Vaginal Birth

Non-clinically indicated CS carries contested and often contradictory meanings that reflect broader gender norms. Popular media in Mexico and New Zealand have framed CS as ‘social caesareans’ or ‘designer deliveries’, invoking ideals of control and elite femininity epitomized by the ‘too posh to push’ trope [39,108]. Here, CS is positioned as a chic, self-affirming choice aligned with popular culture. Within professional discourse, these same cultural logics are often reproduced through terminology like ‘maternal request,’ ‘patient choice,’ or the paradoxical ‘natural caesarean,’ which rhetorically foreground women’s autonomy while simultaneously obscuring the structural and institutional pressures that influence their decisions [39,55,61,73,98,103,105,109].

Conversely, vaginal birth is often imbued with moral significance as a rate of passage that authenticates maternal commitment through endurance of pain [30,52,88,90,95,110]. In focus group with pregnant women in Argentina, Liu et al. (2013) found that the ‘naturalness’ of vaginal delivery was a central theme; as one public-sector participant explained, “*Women have delivered babies like that since the beginning of the history of humankind. Other forms of delivery are man’s inventions*” [30]. Such narratives pull women toward vaginal birth by equating it with authenticity, sacrifice, and maternal virtue.

Importantly, these meanings can change across time, place, and social groups. In higher-income settings, five studies document a generational shift in attitudes toward labor and delivery [22,73,84,95,98]. In a Canadian cohort of 3,680 university students without prior childbirth experience, Stoll et al. (2009) used a mixed-methods online survey to examine childbirth attitudes and preferences [95]. The study found that nearly 9% of participants preferred cesarean delivery, citing fear of labor pain, concerns about bodily damage, and the belief that cesarean birth is safer or healthier [95]. Notably, participants of both sexes viewed elective CS as legitimate – less as a failure of maternal fortitude and more as an exercise of personal agency [95]. These findings underscore a cultural reconfiguration in which feminist self-determination increasingly shapes perceptions of a ‘successful’ birth.

### Sexual Function and Vaginal Integrity

The preservation of vaginal integrity and sexual function emerges as another push factor toward CS, particularly among wealthier populations [35,43,61,80,93]. From France to Singapore, women frequently cite concerns about long-term pelvic floor morbidity – including obstetric anal sphincter injury, urinary or fecal incontinence, dyspareunia, and irreversible perineal trauma – as central motivations for electing cesarean delivery [32,65,83,93,95,106,111]. Vaginal birth was portrayed as an irreversible bodily compromise, despite mixed evidence regarding long-term functional outcomes.

These concerns are closely tied to gender norms that associate feminine worth with bodily integrity, sexual desirability, and partner satisfaction [31,35,43,65,78,84,93,106,111]. Clinical encounters can amplify these beliefs. Counseling practices, risk framing, and selective emphasis on pelvic floor injury may present the vagina as fragile or at risk of permanent damage, which can make CS appear protective [49,65,78,83].

Through in-depth interviews in Phnom Penh, Schantz et al. (2016) document how CS is constructed as a means of preserving sexual desirability [78]. As one Cambodian birth practitioner explained, “*More women want to do the c-section now. Because of the beauty of the perineum… Women think that the fetal head is large and that will enlarge their vagina… It is not aesthetic. And it is not good for the sexuality neither*” [78]. In this context, CS becomes less a clinical intervention than a prophylactic measure to safeguard sexuality, situating birth mode decisions within deeply gendered logics of vaginal preservation.

### Decision-making and autonomy

The literature portrays CS decision-making as a relational process, shaped by layered gendered hierarchies and uneven accountability. Two interrelated sub-themes are particularly salient: first, the role of familial, spousal, and communal authority in structuring women’s reproductive choices; and second, the ways in which women negotiate, appropriate, or resist these constraints, with both cesarean and vaginal birth functioning as sites of empowerment.

### Familial authority and birth mode

The literature documents that decision-making around CS is rarely individualized or evenly shared; rather, it is mediated within complex familial, spousal, and communal networks. In multiple settings, patriarchs exert decisive influence over the timing and mode of delivery, often prioritizing social reputation, perceived safety, or the conservation of household resources at the expense of women’s health needs [23,35,38,54,56,63,64,66,71,81,84,86,102,106,110,112–114]. Evidence from China illustrates this asymmetry: women who initially preferred vaginal birth were more than four times as likely to undergo CS when their husbands favored surgical delivery [23].

In parallel, senior female relatives – namely mothers-in-law – serve as custodians of reproductive knowledge and practice, reinforcing ideals of vaginal delivery [53,67,76,78,81,84]. These pressures go beyond social expectations and can actively restrict women’s decision-making. In a Nigerian antenatal care focus group, one participant described how pregnant women are threatened with marital abandonment should they require CS, with one mother-in-law warning that “*if she...ends up with a CS...that her son won’t be marrying her again as she keeps wasting her son’s money on CS when other women are giving birth by themselves*” [81]. Comparable dynamics appear in qualitative research from Jordan, where women reported that husbands and extended family members actively shaped their delivery decisions, including direct spousal requests for CS based on fears about vaginal birth altering sexual desirability or marital stability [84]. One participant recounted that her husband consulted the physician himself to request surgical delivery because he feared bodily changes associated with vaginal birth [84].

### Constraint and Empowerment

Occasionally, CS functions as a form of negotiated agency, particularly in contexts where maternity services are unpredictable, under-resourced, or inattentive to women’s needs [36,48,72,75]. Drawing on comparative ethnographic research in Brazil, including longitudinal in-depth interviews with 80 postpartum women and interviews with clinicians, Béhague (2002) shows that some women actively appropriated biomedical knowledge, prenatal surveillance, and relationships with specific physicians to negotiate access to cesarean delivery [75]. Rather than passive compliance, operative birth constituted a strategic response to obtain safer, more attentive, and higher-status care within a stratified health system [75]

This negotiated agency is deeply intersectional: Brazilian women with greater economic resources, insurance coverage, and social capital were better positioned to translate preference into access, while those with fewer resources face more limited leverage [75]. Similar patterns emerged in Southeast Asia. Using nationally representative survey data and parity-stratified multivariate analysis, de Loenzien et al. (2021) found that among multiparous Vietnamese women, intrinsic agency – measured as disapproval of intimate partner violence – was associated with more than double the odds of elective CS (OR = 2.42) [36].

Conversely, the literature also documents contexts in which vaginal birth is experienced as the most empowering mode of delivery, particularly where women associate it with bodily integrity, rapid recovery, maternal competence [44,83]. Vaginal birth was also perceived to support relational bonding, the early emotional and psychosocial connection between mother and infant fostered through immediate postnatal contact [44,83]. In a mixed-methods study of childbirth experiences among French women, Schantz et al. (2021) combined closed questionnaires administered to 284 pregnant women with 26 in-depth qualitative interviews across two maternity hospitals in and around Paris [83]. Quantitatively, 97.5% of respondents expressed a preference for vaginal birth, most commonly because it was perceived as more natural (79.1%), less risky (17.7%), and associated with faster recovery and bodily preservation [83].

These findings were reinforced in qualitative interviews, where French women often perceived vaginal birth as “natural” and CS as a technological intervention (“*Instead of a delivery, we have an operation*”) [83]. Several participants explicitly linked vaginal birth to maternal legitimacy and identity, describing it as central to “feeling like a mother,” while cesarean birth was experienced by some as alienating (“*A caesarean section is like you’ve never delivered a baby*”) [83]. Women also articulated biomedical rationales for preferring vaginal birth, including beliefs that it supports neonatal immunity, facilitates breastfeeding, and strengthens early mother–child bonding [83]. For some, empowerment lay not in technological control but in ‘mastering’ physiological childbirth.

## Discussion

This scoping review demonstrates that CS utilization is not solely a clinical outcome based on medical indications, but rather the product of gendered influences operating across multiple, interconnected levels. Across 95 studies and diverse global contexts, CS emerged as a socially embedded practice shaped by household power dynamics, community norms, provider practices grounded in gender norms and expectations, and broader health system structures. Push and pull factors rarely function independently; rather, they interact to produce patterned inequities in access to surgical birth, in women’s capacity to exercise agency, and in how different forms of childbirth are valued within society.

A central theme is that the medicalization of childbirth redistributes authority over reproduction *away* from women and *toward* professional and institutional actors. Childbirth is repeatedly framed as a problem requiring expert management, positioning clinicians as the primary arbiters of risk and intervention. Medical authority becomes normalized as protective, while women’s embodied knowledge or preferences may be implicitly treated as secondary. Such dynamics are consistent with longstanding critiques of medical paternalism, and obstetric care appears particularly susceptible to this model. The literature on obstetric violence suggests that in extreme cases these dynamics manifest as coercive practices surrounding CS, including pressure to consent, dismissal of women’s concerns, and surgical intervention undertaken without meaningful consent [115–117].

At the same time, this review suggest that CS overuse cannot be understood without examining the gendered political economy of maternity care. Financing arrangements often normalize surgical delivery through differential reimbursement, hospital profit structures, and provider convenience that deprioritizes or discounts women’s needs, preferences, or best interests [118]. In settings with weak or overstretched health systems, structural constraints such as inadequate staffing, high patient loads, and limited capacity for continuous labor support may further normalize interventionist care, inadvertently privileging surgical delivery as a means of managing institutional pressures rather than centering women’s needs [119]. These clinical power dynamics mirror wider gender hierarchies in which authority over women’s bodies is routinely externalized to male-dominated institutions and expert actors [120].

Crucially, individual-level motivations appear to be socially constructed rather than purely personal. A global qualitative evidence synthesis by Colomar et al. (2021) found that women’s preferences for cesarean delivery were most strongly influenced by fear of labor pain, concern for maternal or infant injury, and uncertainty surrounding vaginal birth [121]. Our review acknowledges that these fears are not formed in isolation; they are amplified by concerns around body image concerns, sexual desirability, and vaginal recovery. As a result, women’s expressed “preferences” for cesarean delivery often reflect adaptation to structural, interpersonal, and symbolic pressures rather than unconstrained choice.

Conversely, powerful gendered pull factors discourage CS and sustain preferences for vaginal birth in many settings. Community norms valorizing endurance, “naturalness,” and maternal virtue position vaginal birth as a moral achievement and CS as a potential failure of womanhood. Women across contexts often describe vaginal birth as central to maternal identity and social legitimacy, reflecting motherhood as both a social passage and an embodiment of feminine norms [122].

Material conditions further reinforce preferences for vaginal birth, particularly in LMICs. In these contexts, vaginal birth may represent the *only* viable option to sustain household labor, income generation, and childcare responsibilities. In contrast, HICs generally provide greater social and economic support for post-cesarean recovery, making structural barriers to surgical birth less pronounced, though normative and clinical pressures still shape decision-making.

Familial power relations operate ambivalently across contexts: in some LMICs, husbands or elders may encourage cesarean for perceived safety, whereas they may also enforce vaginal birth through threats of abandonment or economic sanction. These dynamics underscore that deterrents to cesarean delivery are not merely cultural preferences but are embedded within gendered systems of labor, dependency, and social accountability.

Taken together, these findings suggest that childbirth operates as a key site where gendered power relations are enacted and negotiated. Both CS and vaginal births can represent forms of agency, but the ability to exercise that agency is unevenly distributed. Women with greater economic resources, social capital, or relationships with providers are often better positioned to shape birth outcomes according to their preferences. For marginalized women, however, reproductive decisions may remain constrained by provider authority, institutional barriers, or familial control. These patterns reflect broader processes of stratified reproduction, in which different groups of women experience distinct reproductive possibilities based on their social position [123].

### Implications for future research

This scoping review also reveals several important gaps in the existing literature. First, despite the review’s deliberate search strategy, race and ethnicity remain strikingly under-examined as independent or interacting determinants of CS utilization. While several studies from the United States, Australia, and Europe allude to migrant status or language barriers, few explicitly analyze how racialization, structural racism, or ethnic marginalization shape access to medically indicated CS, exposure to unnecessary surgical intervention, or experiences of obstetric authority. This absence is notable given well-documented racial disparities in maternal morbidity and mortality and suggests that gendered analyses of CS have not yet fully incorporated race and ethnicity as constitutive axes of power rather than descriptive background variables.

Second, the literature remains heavily weighted toward women’s perceptions and preferences, with comparatively limited attention to the gendered positionality of providers, health system managers, insurers, or policymakers. Although medical paternalism is frequently invoked, relatively few studies empirically examine how clinicians’ gender, class, training, or institutional accountability structures interact with medico-legal risk, professional norms, and financial incentives to produce patterned CS practices. Future research would benefit from shifting analytic focus upstream to examine how gender operates within professional hierarchies, organizational cultures, and regulatory environments that normalize surgical birth.

Third, while many studies acknowledge socioeconomic stratification, intersectional analyses remain underdeveloped. Gender is often examined alongside education, income, or urban–rural residence, but rarely in ways that theorize how these dimensions mutually constitute women’s reproductive agency. For example, adolescent age, marital status, disability, and ethnicity are inconsistently addressed, and LGBTQ+ and gender-diverse birthing people are almost entirely absent from the literature. This limits understanding of how CS decision-making is shaped by intersecting forms of marginalization beyond cisgender womanhood.

Fourth, there is a relative paucity of research examining men’s and partners’ gendered roles beyond their influence as decision-makers. While several studies document spousal authority over birth mode, few interrogate how masculinities, expectations of fatherhood, or economic control intersect with institutional practices to shape CS utilization. This gap constrains the development of interventions that engage partners not merely as gatekeepers but as potential allies in promoting equitable, respectful maternity care.

### Strengths and limitations

This scoping review has several notable strengths. First, it applies a theory-informed gender lens to the study of CS, a domain that has largely been dominated by clinical, epidemiologic, or utilization-focused analyses. By centering gender norms, power relations, and institutional dynamics, the review moves beyond individual preference frameworks to systematically map how cesarean delivery is socially produced across multiple levels of gendered influence. The use of a gender analysis matrix enabled a structured and transparent synthesis of heterogeneous evidence while remaining attentive to the relational and contextual nature of gendered processes.

Second, the review employed a broad and deliberately inclusive search strategy across three major databases, spanning 25 years and capturing qualitative, quantitative, mixed-methods, and policy-oriented studies from more than 30 countries. This breadth allowed the review to surface common gendered mechanisms shaping cesarean delivery across diverse sociopolitical and health system contexts, while also identifying regionally specific dynamics. The inclusion of studies from low-, middle-, and high-income settings strengthens the review’s contribution to global comparative scholarship on surgical birth.

Third, the analytic distinction between gendered push and pull factors, examined across individual, community, and health system levels, represents a conceptual contribution. This framing highlights that overuse and underuse of CS are not opposing phenomena but are produced through overlapping gendered incentives, constraints, and moral economies. By synthesizing these dynamics across the political economy of care, clinical culture, familial authority, and embodied meanings of birth, the review offers a multi-level account that is directly relevant to health systems reform, quality of care, and reproductive justice agendas.

At the same time, some limitations should be acknowledged. As a scoping review, this study does not assess the methodological quality or risk of bias of included studies, and its findings should be interpreted as mapping the contours of the evidence base rather than establishing causal relationships. The literature synthesized is heavily weighted toward cross-sectional designs, which are well suited to examining norms, meanings, and power relations but limit inference about directionality or magnitude of effects. Although the review aimed for global coverage, the geographic distribution of included studies was uneven. A substantial proportion of the evidence derives from a limited set of countries, including Nigeria, Iran, Australia, Sweden, and the United States, while large regions, particularly parts of sub-Saharan Africa and South Asia remain underrepresented. This imbalance reflects broader inequities in global health research production and constrains the generalizability of specific findings, even as cross-cutting gender patterns were observed.

The review is also limited by its language restrictions, as only English- and French-language publications were included. As a result, some region-specific gendered dynamics may be underrepresented. In addition, while intersectionality was a guiding analytic concern, the available evidence rarely enabled robust intersectional analysis. Finally, the process of categorizing findings into analytic domains and levels of influence inevitably involved interpretive judgment. Many studies cut across multiple gender domains simultaneously, and while the matrix approach enhanced consistency, some degree of subjectivity in classification was unavoidable. To mitigate this, extraction and synthesis were conducted iteratively and collaboratively, with cross-checking among reviewers. Despite these limitations, this scoping review provides a comprehensive and conceptually grounded synthesis of how gender operates within CS decision-making and access globally.

## Conclusion

Taken together, the evidence synthesized in this review demonstrates that CS utilization is governed by interacting gendered forces across levels of influence: political–economic structures shape institutional incentives; institutional norms structure provider practices; provider practices shape women’s perceptions of risk and legitimacy; and these perceptions are filtered through household power relations, community norms, and material constraints. This multi-level entanglement challenges technocratic framings of CS overuse and underuse and instead situates surgical birth within broader systems of gendered power. Any response to CS inequities must therefore engage not only clinical guidelines but also the financial architectures of health systems, the hierarchies of obstetric practice, the cultural meanings attached to women’s bodies, and the social conditions that delimit reproductive agency.

## Supporting information

S1 Checklist Preferred Reporting Items for Systematic reviews and Meta-Analyses extension for Scoping Reviews (PRISMA-ScR) checklist. Reproduced from the PRISMA Extension for Scoping Reviews (PRISMA-ScR): Checklist and Explanation, licensed under the Creative Commons Attribution 4.0 International (CC BY 4.0) License.
(DOCX)

S1 Table Search terms. Complete search strategies used for the literature search in PubMed, Embase, and CINAHL, including all search terms, Boolean operators, date limits, and language restrictions.
(PDF)

S2 Table Gender analysis matrix. Gender analysis matrix developed for data extraction and thematic synthesis, organized by gender analysis domains, topic domains, and intersectional considerations.
(PDF)

S3 Table Demographics and results of all included articles (n = 95). (PDF)
