# Supplementary material for: Mapping the gendered dynamics of cesarean section: A scoping review
Source: PLOS Glob Public Health. 2026 Jul 17;6(7):e0006634. doi: 10.1371/journal.pgph.0006634 (PMC13379086; doi:10.1371/journal.pgph.0006634)
Supplement: S1 Table — Complete search strategies used for the literature search in PubMed, Embase, and CINAHL, including all search terms, Boolean operators, date limits, and language restrictions. (PDF) [file pgph.0006634.s002.pdf]

Supplemental 1. Search Terms

Database: PubMed	Search Terms
Concept 1: C-Section	("Cesarean Section"[MeSH] OR "Cesarean Section"[tiab] OR "c-section"[tiab] OR "cesarean"[tiab] OR "caesarean"[tiab] OR "cesarean deliver*"[tiab] OR "caesarean delivery"[tiab] OR "surgical birth*"[tiab] OR "operative deliver*"[tiab] OR "elective cesarean"[tiab] OR "emergency cesarean"[tiab] OR "cesarean section rate*"[tiab] OR "trial of labor"[tiab] OR "trial of labor after cesarean"[tiab] OR "TOLAC"[tiab] OR "vaginal birth after cesarean"[tiab] OR "VBAC"[tiab]) AND ("2000/01/01"[Date - Publication] : "2025/12/31"[Date - Publication]) AND (english[lang] OR french[lang])
Concept 2: Gender	("Sex Factors"[MeSH] OR "Sex Characteristics"[MeSH] OR "sex factor*"[tiab] OR "sex difference*"[tiab] OR "gender norm*"[tiab] OR "gender role*"[tiab] OR "gender bias*"[tiab] OR "gender equity"[tiab] OR "gender inequit*"[tiab] OR "gender equalit*"[tiab] OR "gender inequalit*"[tiab] OR "gender power relation*"[tiab] OR "feminin*"[tiab] OR "patriarch*"[tiab] OR "women's autonomy"[tiab] OR "reproductive autonomy"[tiab] OR "bodily autonomy"[tiab] OR "women's role*"[tiab] OR "gender dynamic*"[tiab]) AND ("2000/01/01"[Date - Publication] : "2025/12/31"[Date - Publication]) AND (english[lang] OR french[lang])
Concept 3: Gendered Influences	("Power, Psychological"[MeSH] OR "Social Stigma"[MeSH] OR "Social Determinants of Health"[MeSH] OR "Informed Consent"[MeSH] OR "Attitude of Health Personnel"[MeSH] OR "Health Knowledge, Attitudes, Practice"[MeSH] OR "Reproductive Rights"[MeSH] OR "Patient Rights"[MeSH] OR "empower*"[tiab] OR "disempower*"[tiab] OR "coerc*"[tiab] OR "time poverty"[tiab] OR "reproductive labour"[tiab] OR "reproductive labor"[tiab] OR "domestic work*"[tiab] OR "sexual norm*"[tiab] OR "sexual desirab*"[tiab] OR "status symbol*"[tiab] OR "modernity"[tiab] OR "stigma"[tiab] OR "abus*"[tiab] OR "mistreat*"[tiab] OR "obstetric violence"[tiab] OR "provider bias*"[tiab] OR "structural barrier*"[tiab] OR "financial incentiv*"[tiab] OR "cost barrier*"[tiab] OR "pathologiz*"[tiab] OR "overmedical*"[tiab] OR "over-medical*"[tiab] OR "birth preference*"[tiab] OR "maternal decision-making"[tiab] OR "provider attitude*"[tiab] OR "institutional norm*"[tiab] OR "medical paternalism"[tiab] OR "medicalization of childbirth"[tiab] OR "reproductive justice"[tiab] OR "maternal right*"[tiab] OR "reproductive right*"[tiab] OR "gender-responsive care"[tiab]) AND ("2000/01/01"[Date - Publication] : "2025/12/31"[Date - Publication]) AND (english[lang] OR french[lang])

**Database: Embase**	**Search Terms**
Concept 1: C-Section	('cesarean section'/de OR 'cesarean section':ti,ab OR 'c-section':ti,ab OR 'cesarean':ti,ab OR 'caesarean':ti,ab OR 'cesarean deliver*':ti,ab OR 'caesarean delivery':ti,ab OR 'surgical birth*':ti,ab OR 'operative deliver*':ti,ab OR 'elective cesarean':ti,ab OR 'emergency cesarean':ti,ab OR 'cesarean section rate*':ti,ab OR 'trial of labor':ti,ab OR 'trial of labor after cesarean':ti,ab OR 'tolac':ti,ab OR 'vaginal birth after cesarean':ti,ab OR 'vbac':ti,ab) AND [2000-2025]/py AND ([english]/lim OR [french]/lim)
Concept 2: Gender	('sex factor'/de OR 'sex characteristic'/de OR 'sex factor*':ti,ab OR 'sex difference*':ti,ab OR 'gender norm*':ti,ab OR 'gender role*':ti,ab OR 'gender bias*':ti,ab OR 'gender equity':ti,ab OR 'gender inequit*':ti,ab OR 'gender equalit*':ti,ab OR 'gender inequalit*':ti,ab OR 'gender power relation*':ti,ab OR 'feminin*':ti,ab OR 'patriarch*':ti,ab OR 'womens autonomy':ti,ab OR 'reproductive autonomy':ti,ab OR 'bodily autonomy':ti,ab OR 'womens role*':ti,ab OR 'gender dynamic*':ti,ab) AND [2000-2025]/py AND ([english]/lim OR [french]/lim)
Concept 3: Gendered Influences	('power psychology'/de OR 'social stigma'/de OR 'social determinant of health'/de OR 'health care personnel attitude'/de OR 'health knowledge attitude practice'/de OR 'reproductive rights'/de OR 'patient rights'/de OR 'empower*':ti,ab OR 'disempower*':ti,ab OR 'coerc*':ti,ab OR 'time poverty':ti,ab OR 'reproductive labour':ti,ab OR 'reproductive labor':ti,ab OR 'domestic work*':ti,ab OR 'sexual norm*':ti,ab OR 'sexual desirab*':ti,ab OR 'status symbol*':ti,ab OR 'modernity':ti,ab OR 'stigma':ti,ab OR 'abus*':ti,ab OR 'mistreat*':ti,ab OR 'obstetric violence':ti,ab OR 'provider bias*':ti,ab OR 'structural barrier*':ti,ab OR 'financial incentiv*':ti,ab OR 'cost barrier*':ti,ab OR 'pathologiz*':ti,ab OR 'overmedical*':ti,ab OR 'over-medical*':ti,ab OR 'informed consent':ti,ab OR 'birth preference*':ti,ab OR 'maternal decision-making':ti,ab OR 'provider attitude*':ti,ab OR 'institutional norm*':ti,ab OR 'medical paternalism':ti,ab OR 'medicalization of childbirth':ti,ab OR 'reproductive justice':ti,ab OR 'maternal right*':ti,ab OR 'reproductive right*':ti,ab OR 'gender-responsive care':ti,ab) AND [2000-2025]/py AND ([english]/lim OR [french]/lim)

**Database: CINAHL***	**Search Terms**
Concept 1: C-Section	("c-section" OR "cesarean" OR "caesarean" OR "cesarean deliver*" OR "caesarean delivery" OR "surgical birth*" OR "operative deliver*" OR "elective cesarean" OR "emergency cesarean" OR "cesarean section rate*" OR "trial of labor" OR "trial of labor after cesarean" OR "TOLAC" OR "vaginal birth after cesarean" OR "VBAC")
Concept 2: Gender	("sex factor*" OR "sex difference*" OR "gender norm*" OR "gender role*" OR "gender bias*" OR "gender equity" OR "gender inequit*" OR "gender equalit*" OR "gender inequalit*" OR "gender power relation*" OR "feminin*" OR "patriarch*" OR "women's autonomy" OR "reproductive autonomy" OR "bodily autonomy" OR "women's role*" OR "gender dynamic*")
Concept 3: Gendered Influences	("empower*" OR "disempower*" OR "coerc*" OR "time poverty" OR "reproductive labour" OR "reproductive labor" OR "domestic work*" OR "sexual norm*" OR "sexual desirab*" OR "status symbol*" OR "modernity" OR "stigma" OR "abus*" OR "mistreat*" OR "obstetric violence" OR "provider bias*" OR "structural barrier*" OR "financial incentiv*" OR "cost barrier*" OR "pathologiz*" OR "overmedical*" OR "over-medical*" OR "birth preference*" OR "maternal decision-making" OR "provider attitude*" OR "institutional norm*" OR "medical paternalism" OR "medicalization of childbirth" OR "reproductive justice" OR "maternal right*" OR "reproductive right*" OR "gender-responsive care")

*Manually limited to English Or French publications after 2000
