# Supplementary material for: Mapping the gendered dynamics of cesarean section: A scoping review
Source: PLOS Glob Public Health. 2026 Jul 17;6(7):e0006634. doi: 10.1371/journal.pgph.0006634 (PMC13379086; doi:10.1371/journal.pgph.0006634)
Supplement: S2 Table — Gender analysis matrix developed for data extraction and thematic synthesis, organized by gender analysis domains, topic domains, and intersectional considerations. (PDF) [file pgph.0006634.s003.pdf]

Supplemental 2. Gender Analysis Matrix

Topic Domains	Intersectional Considerations	Gender Analysis Domains				
		Access to resources	Roles and practices	Norms, values, beliefs	Decision-making and autonomy	Laws, policies, institutions
**Push factors: what encourages people to pursue C-section**	Intersectional patterns: Education, urban residence, private-sector access, and higher socioeconomic status are repeatedly associated with greater C-section uptake and with framing C-section as prestige or empowerment; younger age (adolescents) may be more deferential to medical authority and thus more likely to accept provider-driven C-section.	Financial drivers<br><br>**Provider and private-sector financial incentives**: physician income, hospital profit motives, and payment structures (e.g., misaligned patient co-payments in non-public hospitals) make C-sections more lucrative and encourage higher rates in private settings.<br><br>**Socioeconomic stratification of access**: Higher socioeconomic status and private-sector access often correlate with greater C-section uptake (social prestige/class signaling and ability to pay); gendered economic advantages.<br><br>Information & technology<br><br>**Differential information access**: Internet use and education increase women's exposure to biomedical narratives that sometimes normalize C-section as modern/safer, facilitating elective uptake among more educated or urban women.<br><br>Human resources & facility resources<br><br>**Institutional capacity that favors surgical solutions**: Facilities with surgical capacity, staffing patterns and predictable operating schedules render C-section more available and often more readily offered than prolonged vaginal management.	Clinical routines and provider practices<br><br>**Physician convenience and routine scheduling**: C-sections are promoted where provider routines, convenience, and predictability are prioritized, making C-section a normalized clinical practice.<br><br>**Delegation away from midwifery models**: Institutional practices that reduce continuous labor support (staffing shortages, poor labor monitoring) shift birth management toward operative solutions.<br><br>**Provider gender:** male obstetricians were described as more likely to favor surgical control, while female obstetricians emphasized relational continuity, though both groups reinforced norms framing C-section as socially acceptable<br><br>Household, caregiving, and femininity roles<br><br>**Women's labor and caretaking constraints**: Practical household roles and anticipated caregiving responsibilities (e.g., need for quick recovery to resume domestic work) are reported as motivators for choosing C-section in some contexts.<br><br>**Social performance of femininity:** For some women, elective C-section is a practiced role aligned with maintaining appearance, composure, and normative ideals of motherhood<br><br>Brokerage and intermediaries<br><br>**Influence of intermediaries**: often leverage women's limited autonomy and social dependence, motivated by financial incentives and reinforcing gendered norms of status, safety, and modernity.	Cultural meanings attached to modes of birth<br><br>**C-section as modernity, status, or protection**: C-sections are framed as modern, safe, or prestigious – symbols of socioeconomic status and modern femininity – encouraging uptake among those seeking these social meanings.<br><br>**Fear and avoidance of vaginal birth**: Widespread narratives of vaginal birth as painful, risky, or degrading (including "horror stories") produce fear that drives demand for C-section as a perceived safer or more controllable option.<br><br>**Son preference:** Cultural valuation of male offspring increases willingness of families and providers to request or offer C-section for male fetuses –especially at first birth – reflecting gendered norms about lineage, status, and reproductive value.<br><br>Gendered aesthetic and sexual concerns<br><br>**Body image and sexual satisfaction anxieties**: Concerns about sexual function or body integrity following vaginal birth motivate some women (or partners) to prefer C-section.	Deference and medical authority<br><br>**Deference to physician authority**: Some women defer to provider recommendations; where providers recommend C-section, deference (amplified by trust or paternalism) increases uptake.<br><br>Family and partner roles<br><br>**Joint or partner-led decisions can encourage C-section**: Joint spousal decision-making or male partner influence aligns with choices favoring C-section (sometimes framed as protecting mother/child, preserving status, or preventing labor risks). This shifts decision-making authority toward external/family influence rather than clinical indication.<br><br>Strategic use of medicalization<br><br>**Medicalization as an empowerment tactic**: Some women (particularly those with resources) actively choose C-section as a strategic pathway to exercise agency within constrained social systems – using the procedure to navigate class, professional, or personal concerns.	Health system incentives and organization<br><br>**Institutional financial and organizational drivers**: Payment systems and private sector incentives, together with hospital routines that privilege surgical throughput (e.g., limited presence of relatives, limited pain relief), structurally encourage C-sections.<br><br>Health policy<br><br>**Weak governance over non-indicated C-sections**: Policy narratives supporting maternal autonomy – without parallel emphasis on balanced counseling – create environments where maternal-request C-section is read as fully legitimate without systematic assessment of risks.<br><br>**Medico-legal environment:** Male-dominated clinical and institutional cultures may amplify defensive practices, with providers – especially in hierarchical settings – favoring C-section to minimize liability/litigation, reinforcing interventionist norms and limiting patient-centered decision-making.

**Pull factors: what encourages people *not* to pursue C-section**	Intersectional vulnerabilities: rural residence, low socioeconomic status, younger age (context dependent), lower education, and marginalized ethnic or social group membership appear as amplifiers of restricted access and reduced autonomy to pursue medically necessary C-sections.	Financial barriers<br><br>**High out-of-pocket costs**: High financial costs of C-section in private facilities and inadequate financial protection restrict access for poorer women; patient co-payments and lack of affordability are repeatedly cited deterrents.<br><br>**Household spending and prioritization**: Intra-household health-spending norms often prioritize men's health or other family needs over women's obstetric care.<br><br>Geographic and infrastructure inequities<br><br>**Poor distribution of emergency obstetric care**: Rural and resource-poor areas show limited availability of surgical services/resources (shortage of midwives, poor facilities), producing access gaps for clinically indicated C-sections.<br><br>Information deficits<br><br>**Lack of informed decision-making**: Low education, limited antenatal education, and poor information flow mean women may not be aware of indications or avenues for surgical care, delaying or preventing access.	Caregiving expectations and labor roles<br><br>**Domestic role obligations**: Responsibilities for childcare and household labor can deter women from seeking or consenting to C-section when recovery is expected to disrupt caregiving roles.<br><br>Provider attitudes and discriminatory practices<br><br>**Public hospital neglect and coercive practices**: Studies highlight neglect, mistreatment, or coercion in public settings – practices that discourage women from engaging with facilities or consenting to recommended surgical care.<br><br>Familial gatekeeping<br><br>**Influence of elders and in-laws**: Family elders' authority and collective decision practices (in-laws, extended family) can discourage C-section in favor of traditional/vaginal birth norms.	Cultural valorization of vaginal birth<br><br>**Endurance and womanhood narratives**: Cultural norms valorizing endurance in childbirth and "natural" delivery can stigmatize C-sections and deter women from pursuing them even when medically indicated, including in contexts where emergency C-sections carry high morbidity.<br><br>**Public norms of medical justification:** In some settings, public opinion accepts maternal request only when medical reasons are present; social norms that view fear or desire alone as insufficient justification act as deterrents to elective C-section<br><br>Religious/spiritual framing<br><br>**Religious or cultural prohibition**: In some settings religious or spiritual beliefs discourage surgical birth or frame intervention as inappropriate, limiting acceptance of C-section.<br><br>Moral critique of overmedicalization<br><br>**Rejection of medicalization by some groups**: Elite or socially critical women may resist C-section as symbolic of unnecessary medicalization, producing a counter-movement that can lower elective and, in some cases, necessary uptake.	Male and familial authority limiting access<br><br>**Male dominance and required consent**: Male authority and patriarchal consent norms (husband/partner or elders making final decisions) can delay or refuse approval for C-section, particularly where family members fear stigma or financial burden. Some male partners discourage facility births, framing hospital delivery (and therefore access to C-section) as unnecessary or financially wasteful, especially in low-resource settings.<br><br>Women's limited autonomy<br><br>**Low autonomy, low education**: Women with limited autonomy and lower education levels are less able to advocate for medically indicated surgical care, and may be excluded from decision-making.<br><br>Fear of social judgment<br><br>**Perceived moral judgment**: Women who anticipate moral stigma or blame for requesting C-section may avoid pressing for needed care.	Systemic neglect and under-resourcing<br><br>**Weak emergency obstetric systems**: Institutional deficits (staff shortages, lack of midwives, limited operating capacity in public sector) and uneven geographic distribution of services act as structural deterrents to timely C-section access. Policies emphasizing vaginal birth without sufficient investment in supportive care infrastructure (midwifery, analgesia, respectful care) can unintentionally constrain access to necessary C-section<br><br>Policy and payment structures that disadvantage the poor<br><br>**Absence of protective financing or referral systems**: Where payment systems and referral pathways are inadequate, low-income women face systemic barriers to accessing C-sections when indicated.<br><br>Professional norms and clinical cultures<br><br>**Clinical cultures that deprioritize patient participation**: Institutionalized paternalism and provider gatekeeping may produce delayed referral or refusal of C-section for marginalized women.<br><br>**Institutional distrust and perceived poor quality constrain access:** Low trust in healthcare – rooted in perceived maltreatment or poor quality – shapes public attitudes and may deter women from engaging with services where C-section would be indicated or negotiated. Weak enforcement of respectful maternity-care standards contributes to mistreatment fears that deter timely facility use and undermine women's willingness to undergo C-section.
