# Supplementary material for: Mapping the gendered dynamics of cesarean section: A scoping review
Source: PLOS Glob Public Health. 2026 Jul 17;6(7):e0006634. doi: 10.1371/journal.pgph.0006634 (PMC13379086; doi:10.1371/journal.pgph.0006634)
Supplement: S3 Table — (PDF) [file pgph.0006634.s004.pdf]

Supplemental Table 3. Demographics and results of all included articles (n=95)

Citation	Title	Year	Setting	Relevant Sub-Themes and Findings
Aali BS and Motamedi B. "Women's Knowledge and Attitude towards Modes of Delivery in Kerman, Islamic Republic of Iran." East Mediterr Health J (Egypt) 11, no. 4 (2005): 663–72.	Women's knowledge and attitude towards modes of delivery in Kerman, Islamic Republic of Iran.	2005	Iran	**Stratified Access to Surgical Birth:** Preference for CS was strongly conditioned by economic capacity—34.3% of women preferred CS only in the absence of economic problems, and housewives expressed stronger preference for vaginal birth, reflecting constrained access to household financial resources for women's health care.<br><br>**Managing Birth Pain and Fear:** Perceptions of pain and risk were central to preferences: 31.4% viewed CS as less painful than vaginal delivery, while an equal proportion preferred vaginal birth due to fear of anesthesia, illustrating gendered anxieties shaped by limited pain management options and surgical mistrust.<br><br>**Social Meanings of Cesarean and Vaginal Birth:** Vaginal delivery was overwhelmingly moralized as "natural" (96.5%), while 40.5% held negative attitudes toward CS; these views were reinforced by community norms valuing maternal endurance and beliefs that young women's bodies are biologically inadequate for vaginal birth.
Adeniran AS, Ogunlaja OO, Ogunlaja IP, et al. "Pre-and-Post-Operative Aversion among Men Whose Partners Had Caesarean Delivery in a Patriarchal Setting." Ghana Med J (Ghana) 55, no. 4 (2021): 285–91. https://doi.org/10.4314/gmj.v55i4.9.	Pre-and-post-operative aversion among men whose partners had caesarean delivery in a patriarchal setting.	2021	Nigeria	**Familial Authority and Birth Mode:** Male partners exercised decisive control over emergency CS—52.7% stated that consent is the man's right, while only 4.0% agreed women could consent themselves, resulting in delayed access to surgery even in life-threatening situations.<br><br>**Managing Birth Pain and Fear:** Pre-operative aversion was driven by fear of mortality and reproductive loss—30.0% feared maternal death and 34.7% believed CS would limit future fertility—yet aversion substantially decreased post-operatively after men observed positive maternal and neonatal outcomes.<br><br>**Stratified Access to Surgical Birth:** Cost was a major deterrent, with 60.7% of men perceiving CS as expensive, highlighting how lack of financial protection intersects with patriarchal consent norms to constrain women's timely access to emergency obstetric care.
Adewuyi EO, Akosile W, Olutuase V, et al. "Caesarean Section and Associated Factors in Nigeria: Assessing Inequalities between Rural and Urban Areas-Insights from the Nigeria Demographic and Health Survey 2018." BMC Pregnancy Childbirth (England) 24, no. 1 (2024): 538. https://doi.org/10.1186/s12884-024-06722-6.	Caesarean section and associated factors in Nigeria: assessing inequalities between rural and urban areas-insights from the Nigeria Demographic and Health Survey 2018.	2024	Nigeria	**Stratified Access to Surgical Birth:** CS utilization in Nigeria was extremely low overall (2.7%) and nearly fourfold lower in rural areas (1.2%) than urban areas (5.2%), with wealth and maternal education strongly increasing CS odds across settings, underscoring socioeconomic and geographic inequities in access to life-saving obstetric care.<br><br>**Familial Authority and Birth Mode:** Women who reported no difficulty obtaining permission for healthcare and those engaged in joint spousal decision-making (particularly in rural areas) had significantly higher odds of CS, indicating that relational autonomy and male partner involvement can facilitate access rather than constrain it.<br><br>**Constraint and Empowerment:** Indicators of empowerment—including higher maternal education, husbands' education (rural), optimal ANC attendance (≥8 visits), and internet use (urban)—were all associated with increased CS odds, while low education, poverty, and restrictive religious or cultural norms functioned as structural deterrents to utilization.

Amyx M, Gibbons L, Xiong X, et al. "Sources of Influence on Pregnant Women's Preferred Mode of Delivery in Buenos Aires, Argentina." Birth (United States) 45, no. 1 (2018): 71–78. https://doi.org/10.1111/birt.12307.	Sources of influence on pregnant women's preferred mode of delivery in Buenos Aires, Argentina.	2019	Argentina	**Physician Gender and Medical Paternalism:** Delivery mode preference was heavily influenced by doctors/midwives, partners/spouses, and mothers, with public-sector women more likely to defer to physicians as final decision-makers, reflecting power imbalances in patient-provider dynamics.<br><br>**Familial Authority and Birth Mode:** Younger women and those of lower SES were more influenced by family and media sources (magazines, TV/movies), highlighting the role of social networks and socio-economic position in shaping perceived authority over childbirth decisions.<br><br>**Constraint and Empowerment:** Knowledge of cesarean indications was moderate and varied by socio-demographics, but sources influencing preference had little impact on actual knowledge, suggesting that information exposure does not automatically translate into informed decision-making.
Awoyinka BS, Ayinde OA, and Omigbodun AO. "Acceptability of Caesarean Delivery to Antenatal Patients in a Tertiary Health Facility in South-West Nigeria." J Obstet Gynaecol (England) 26, no. 3 (2006): 208–10. https://doi.org/10.1080/01443610500508311.	Acceptability of caesarean delivery to antenatal patients in a tertiary health facility in south-west Nigeria.	2006	Nigeria	**Commodification and Medicalization of Labor:** Most antenatal patients (majority with tertiary education) accepted caesarean section as a medically indicated mode of delivery, rejecting the notion that it signified failure of their reproductive role.<br><br>**Social Meanings of Cesarean and Vaginal Birth:** While most participants accepted caesarean delivery, a minority attributed it to spiritual causes or punishment for marital infidelity, highlighting persistent cultural and superstitious beliefs.
Aziken M, Omo-Aghoja L, and Okonofua F. "Perceptions and Attitudes of Pregnant Women towards Caesarean Section in Urban Nigeria." Acta Obstet Gynecol Scand (United States) 86, no. 1 (2007): 42–47. https://doi.org/10.1080/00016340600994950.	Perceptions and attitudes of pregnant women towards caesarean section in urban Nigeria.	2007	Nigeria	**Commodification and Medicalization of Labor**: 59% of women were willing to accept caesarean section if medically indicated, rising to 81% when their own or their infant's life was at risk, showing safety as the primary driver of acceptance.<br><br>**Familial Authority and Birth Mode**: Cultural and social pressures—including fear of ridicule, husband's disapproval, and perception of CS as a "failure"—accounted for 32.8% of reasons women resisted CS.<br><br>**Constraint and Empowerment:** Low understanding of CS and beliefs in supernatural causes of labor complications limited acceptance, highlighting the need for targeted antenatal and community-based education to improve knowledge, attitudes, and empowerment around surgical birth.

Bagheri, A., N. Masoudi Alavi, and F. Abbaszadeh. “Iranian Obstetricians’ Views about the Factors That Influence Pregnant Women’s Choice of Delivery Method: A Qualitative Study.” Women and Birth 26, no. 1 (2013): e45–49. https://doi.org/10.1016/j.wombi.2012.09.004.	Iranian obstetricians' views about the factors that influence pregnant women's choice of delivery method: A qualitative study	2013	Iran	**Managing Birth Pain and Fear:** Women’s fear of labor pain and prior negative birth experiences drove preference for elective CS; obstetricians reported that many patients were unprepared for informed decision-making, highlighting limited agency and need for expanded pain management (e.g., epidurals).<br><br>**Social Meanings of Cesarean and Vaginal Birth:** CS is valorized as safer, modern, and prestigious; social and class markers, imitation of physician and educated role models, and media narratives normalize CS, while vaginal delivery is culturally undervalued.<br><br>**Physician Gender and Medical Paternalism:** Systemic factors—including legal vulnerability, staffing shortages, lack of emotional support, absence of institutional incentives for vaginal birth, and physician convenience/income motives—push predominantly female obstetricians toward performing CS, reinforcing high national CS rates.
Bawadi H, Al-Hamdan Z, Shaqra NA, et al. “An Interpretative Phenomenological Study about Maternal Perceptions of Cesarean Birth.” PLoS One (United States) 20, no. 2 (2025): e0318525. https://doi.org/10.1371/journal.pone.0318525.	An interpretative phenomenological study about maternal perceptions of cesarean birth.	2025	Jordan	**Managing Birth Pain and Fear:** Most participants perceived CS as safer and less painful than normal vaginal delivery (NVD), often citing fear of labor pain or past family birth trauma as decisive factors in opting for CS.<br><br>**Sexual Function and Vaginal Integrity:** Concerns about vaginal laxity and potential loss of sexual satisfaction influenced women’s preference for CS; peer networks and generational attitudes also shaped decision-making, with younger mothers favoring CS while older family members encouraged NVD.<br><br>**Physician Gender and Medical Paternalism:** Women’s decisions were strongly guided by physician advice, reflecting structural paternalism, though some participants asserted autonomy, emphasizing self-assurance and personal conviction in choosing CS; financial incentives for doctors were also reported as influencing recommendations.
Bayes S, Fenwick J, and Hauck Y. “‘Off Everyone’s Radar’: Australian Women’s Experiences of Medically Necessary Elective Caesarean Section.” Midwifery (Scotland) 28, no. 6 (2012): e900-9. https://doi.org/10.1016/j.midw.2012.01.004.	'Off everyone's radar': Australian women's experiences of medically necessary elective caesarean section.	2012	Australia	**Commodification and Medicalization of Labor:** Medically necessary elective C-sections were experienced as highly medicalized and procedural; women felt like “just another case” and assumed passive compliance roles, internalizing gendered norms of quietness and stillness during surgery.<br><br>**Constraint and Empowerment:** Exclusion from decision-making and marginalization during surgery led to emotional trauma, feelings of invisibility and humiliation, and potential peritraumatic dissociation, undermining maternal agency and mother–infant bonding postnatally.<br><br>**Social Meanings of Cesarean and Vaginal Birth:** Institutional routines prioritized clinical efficiency over women’s participation; women’s expectations for active involvement and immediate maternal role fulfillment were largely unmet, reflecting structural disempowerment even in medically indicated procedures.

Begum T, Ellis C, Sarker M, et al. "A Qualitative Study to Explore the Attitudes of Women and Obstetricians towards Caesarean Delivery in Rural Bangladesh." BMC Pregnancy Childbirth (England) 18, no. 1 (2018): 368. https://doi.org/10.1186/s12884-018-1993-9.	A qualitative study to explore the attitudes of women and obstetricians towards caesarean delivery in rural Bangladesh.	2018	Bangladesh	Commodification and Medicalization of Labor: In rural Bangladesh, provider financial incentives and influence of brokers contributed to high rates of non-medically indicated C-sections in private hospitals (costs \$282–\$320 vs. \$77–\$128 in public hospitals), while inadequate staffing and poor labor monitoring further pushed surgical birth.<br><br>**Social Meanings of Cesarean and Vaginal Birth:** Women's preferences favored vaginal birth due to cultural norms valuing endurance, family elders' authority, and concerns about post-surgical weakness, but social narratives linking C-sections to modernity and safety created mixed perceptions.<br><br>**Constraint and Empowerment:** Women generally deferred decision-making to healthcare providers and family elders, with widespread misconceptions about C-sections (e.g., "episiotomy as small C-section") and limited understanding of indications, highlighting structural barriers to informed consent and autonomous choice.
Béhague DP. "Beyond the Simple Economics of Cesarean Section Birthing: Women's Resistance to Social Inequality." Cult Med Psychiatry (Netherlands) 26, no. 4 (2002): 473–507. https://doi.org/10.1023/a:1021730318217.	Beyond the simple economics of cesarean section birthing: women's resistance to social inequality.	2002	Brazil	**Constraint and Empowerment:** Poor and marginalized women strategically used medicalized birthing practices, including caesarean section, to secure quality care, avoid mistreatment, and negotiate socioeconomic and gender inequalities, effectively leveraging medicalization as a form of empowerment.<br><br>**Social Meanings of Cesarean and Vaginal Birth:** Cultural and moral pressures stigmatized "deviant" women (teenagers, high-parity, or poor) for normal birth, while elite women often rejected medicalization to assert autonomy and control over their birthing experience.
Béhague DP, Victora CG, and Barros FC. "Consumer Demand for Caesarean Sections in Brazil: Informed Decision Making, Patient Choice, or Social Inequality? A Population Based Birth Cohort Study Linking Ethnographic and Epidemiological Methods." BMJ (England) 324, no. 7343 (2002): 942–45. https://doi.org/10.1136/bmj.324.7343.942.	Consumer demand for caesarean sections in Brazil: informed decision making, patient choice, or social inequality? A population based birth cohort study linking ethnographic and epidemiological methods.	2002	Brazil	**Stratified Access to Surgical Birth:** In Brazil, C-section access was highly stratified by social class—higher-class women were routinely offered or could demand C-sections, whereas poor women were often induced or discouraged from surgical birth. In the cohort ($n=5,304$), women with more household autonomy were 2.4 times more likely to obtain a C-section ($p=0.008$).<br><br>**Social Meanings of Cesarean and Vaginal Birth:** C-sections were culturally framed as markers of modernity, responsible motherhood, and "good quality care," while vaginal birth was stigmatized as risky, painful, and morally suspect, particularly for teenagers, high-parity, or low-income women.<br><br>**Constraint and Empowerment:** Poor women's demand for C-sections reflected rational responses to systemic neglect, coercion, and moralized judgments in public hospitals; institutional bias, privatization, and medical gatekeeping shaped who could access medicalized birth, revealing that C-section rates were driven as much by social inequality as by medical necessity.

Ünlü Bidik, N., and E. Ceber Turfan. 2024. “Perceptions of Women in Turkey on the Concepts of ‘Caesarean Section’ and ‘Vaginal Birth After Caesarean Section’: A Metaphor Analysis.” Bangladesh Journal of Medical Science 23 (2): 418–28. https://doi.org/10.3329/bjms.v23i2.72155.	Perceptions of Women in Turkey on the Concepts of “Caesarean Section” and “Vaginal Birth After Caesarean Section”: A Metaphor Analysis	2024	Turkey	**Managing Birth Pain and Fear:** Women expressed strong fear of vaginal birth, viewing CS as a “lifesaver” that prevents labor pain and protects the baby, though many also described post-surgical pain and difficulty caring for their newborn, indicating mixed perceptions of safety versus trauma.<br><br>**Social Meanings of Cesarean and Vaginal Birth:** CS was perceived as controlled, modern, and socially normative, while vaginal birth after cesarean (VBAC) was seen as risky and uncertain; cultural and societal expectations strongly shaped women’s decision-making and emotional responses.<br><br>**Constraint and Empowerment:** Some women experienced disruption in maternal bonding and a sense of incompleteness after CS, highlighting how systemic medical practices and social norms can constrain women’s agency even when surgery is medically indicated.
Brown E, Carroll J, Fogarty C, and Holt C. “‘They Get a C-Section…They Gonna Die’: Somali Women’s Fears of Obstetrical Interventions in the United States.” J Transcult Nurs (United States) 21, no. 3 (2010): 220–27. https://doi.org/10.1177/1043659609358780.	"They get a C-section...they gonna die": Somali women's fears of obstetrical interventions in the United States.	2010	United States	**Managing Birth Pain and Fear:** 75% of Somali women interviewed (n=34) expressed strong fear of death from C-section, perceiving prolonged labor or surgical intervention as life-threatening based on prior experiences in Africa.<br><br>**Familial Authority and Birth Mode:** Decisions about C-section were heavily influenced by social and familial expectations; women would refuse surgery if family support was unavailable, reflecting the intersection of caregiving responsibilities and limited autonomy.<br><br>**Physician Gender and Medical Paternalism:** Distrust in provider motives was common, with participants perceiving clinicians as financially incentivized to perform C-sections and as rushing labor unnecessarily, highlighting the importance of culturally competent care and negotiated birth plans.
Cappell J and Pukall CF. “Perceptions of the Effects of Childbirth on Sexuality among Nulliparous Individuals.” Birth (United States) 45, no. 1 (2018): 55–63. https://doi.org/10.1111/birt.12321.	Perceptions of the effects of childbirth on sexuality among nulliparous individuals.	2018	Canada & United States	**Sexual Function and Vaginal Integrity:** Among 1,428 nulliparous participants, a subset endorsed beliefs that vaginal birth harms future sexual function, while cesarean delivery is protective; negative attitudes toward female genitalia and heteronormative sexual expectations (“coital imperative”) strongly predicted these beliefs.<br><br>**Social Meanings of Cesarean and Vaginal Birth:** Media exposure—including reality TV, non-reality shows, and online sources—was significantly associated with endorsement of sexual-harm beliefs, whereas participants who rated healthcare professionals as influential sources were less likely to hold such beliefs (PEMDS scores decreased with each higher trust rating).<br><br>**Constraint and Empowerment**: Lack of proactive provider counseling on sexuality postpartum allows misinformation to persist, shaping preferences for elective C-section even absent medical indications; North American cultural norms were associated with slightly lower C-section preference overall.

Carvalho de Matos, Greice, Ana Paula de Lima Escobal, Josiane Santos Palma, Kamila Dias Gonçalves, Evelin Braatz Blank, and Marilu Correa Soares. "Normal or Cesarean Birth In Adolescence: Who Is the Decision?" Journal of Nursing UFPE 12 (June 2018). https://doi.org/10.5205/1981-8963-v12i6a231069p1681-1687-2018.	Normal or cesarean birth in adolescence: who is the decision?	2018	Brazil	**Physician Gender and Medical Paternalism:** Among 30 adolescent participants, delivery decisions were largely concentrated in healthcare professionals' hands, with medical authority driving high cesarean rates and leaving young women with minimal input.<br><br>**Managing Birth Pain and Fear:** Adolescents' fear and lack of knowledge about childbirth led them to defer decisions to providers; women who accessed prenatal education and information demonstrated active decision-making regarding mode of delivery.<br><br>**Familial Authority and Birth Mode:** Decision-making was shaped by multiple sources—family, friends, media, and cultural narratives—including perceptions of vaginal birth as painful and cesarean as safe, illustrating how community and cultural norms intersect with medical authority.
Chadwick, Rachelle Joy, and Don Foster. "Technologies of Gender and Childbirth Choices: Home Birth, Elective Caesarean and White Femininities in South Africa." Feminism & Psychology 23, no. 3 (2013): 317–38. https://doi.org/10.1177/0959353512443112.	Technologies of gender and childbirth choices: Home birth, elective caesarean and white femininities in South Africa.	2012	South Africa	**Managing Birth Pain and Fear:** All 21 participants reported fear or "mortification" at the thought of vaginal birth, leading them to choose elective C-section as a means to maintain control over pain, timing, and bodily autonomy.<br><br>**Social Meanings of Cesarean and Vaginal Birth:** Elective C-sections were framed as a way to perform normative white femininity—preserving appearance, composure, and maternal ideals—showing childbirth choices are shaped by socio-cultural gender scripts, not just medical necessity.<br><br>**Care Economy and Convenience:** Access to elective C-sections was mediated by high-cost private facilities; socio-economic privilege determined who could realistically exercise choice, highlighting inequalities in birthing options within South Africa.
Chong ES and Mongelli M. 2003. "Attitudes of Singapore Women toward Cesarean and Vaginal Deliveries." Int J Gynaecol Obstet (United States) 80 (2): 189–94. https://doi.org/10.1016/s0020-7292(02)00391-0.	Attitudes of Singapore women toward cesarean and vaginal deliveries.	2003	Singapore	**Managing Birth Pain and Fear:** The primary driver for elective cesarean preference was avoidance of labor pain and stress ("tokophobia"), though less than 4% of the 160 women surveyed preferred C-section without medical indication.<br><br>**Sexual Function and Vaginal Integrity:** Preservation of sexual function and urinary continence was cited by only 24% of women; the majority valued vaginal delivery as natural, safer, and less costly, indicating strong cultural and personal preference for vaginal birth.<br><br>**Constraint and Empowerment:** Public sector obstetricians historically did not consider maternal request a valid indication, but suggest that introducing elective C-section policies would moderately increase rates while enhancing maternal satisfaction through shared decision-making.

Cindoglu D and Sayan-Cengiz F. “Medicalization Discourse and Modernity: Contested Meanings over Childbirth in Contemporary Turkey.” Health Care Women Int (England) 31, no. 3 (2010): 221–43. https://doi.org/10.1080/07399330903042831.	Medicalization discourse and modernity: contested meanings over childbirth in contemporary Turkey.	2010	Turkey	**Physician Gender and Medical Paternalism:** Women tolerate authoritarian and degrading treatment from physicians because hospital birth is framed as “modern and respectable,” while physicians prefer predictable C-sections to avoid ambiguity, reinforcing medicalized control.<br><br>**Familial Authority and Birth Mode:** Patrilineal norms and male family authority shape decision-making; women defer to fathers-to-be and view their bodies as vessels for the paternal “seed,” prioritizing the father’s lineage over their own preferences or comfort.<br><br>**Social Meanings of Cesarean and Vaginal Birth**: Birth is socially constructed as risky and requiring institutional management; hospital births symbolize modernity, while home births are stigmatized as “primitive,” driving high rates of medicalized delivery despite women’s dissatisfaction.
Coates D, Donnolley N, Thirukumar P, Lainchbury A, Spear V, and Henry A. “Women’s Experiences of Decision-Making and Beliefs in Relation to Planned Caesarean Section: A Survey Study.” Aust N Z J Obstet Gynaecol (Australia) 61, no. 1 (2021): 106–15. https://doi.org/10.1111/ajo.13255.	Women's experiences of decision-making and beliefs in relation to planned caesarean section: A survey study.	2021	Australia	**Managing Birth Pain and Fear:** 62% of respondents cited fear of repeating a prior traumatic birth (emergency C-section or prolonged labor) as a primary reason for requesting a planned C-section, highlighting trauma and mental health (PTSD, assault history) as central drivers of decision-making.<br><br>**Physician Gender and Medical Paternalism:** Women’s perceptions of safety and control were influenced by provider recommendations and insufficient information-sharing; many reported lacking written guidance about C-sections compared with vaginal birth, limiting informed decision-making.<br><br>**Social Meanings of Cesarean and Vaginal Birth:** Cultural norms framing vaginal birth as “natural” or a “rite of passage” create both pressure and stigma; women balance personal autonomy against expectations from partners, family, and broader societal narratives when choosing birth mode.
Dai, C., X. Wang, C. Xu, L. Bai, and Y. Li. “A Study on the Psychological Experience and Influential Factors of Pregnant Women Who Decided Elective Caesarean Section After Public Health Emergencies – A Cross-Sectional Survey.” International Journal of Women’s Health 15, no. (Dai C.; Wang X.; Xu C., xuchenying@51mch.com; Bai L.) Operating Room, Shanghai Key Laboratory of Maternal Fetal Medicine, Shanghai Institute of Maternal-Fetal Medicine and Gynecologic Oncology, Shanghai First Maternity and Infant Hospital, School of Medi (2023): 1713–25. https://doi.org/10.2147/IJWH.S418959.	A Study on the Psychological Experience and Influential Factors of Pregnant Women Who Decided Elective Caesarean Section After Public Health Emergencies ‚Äì A Cross-Sectional Survey	2023	China	**Managing Birth Pain and Fear:** 595 women who chose elective C-sections after COVID-19 reported measurable anxiety (GAD-7) and lower general well-being; psychological readiness was strongly associated with prior pregnancy complications, number of abortions, and personal education/income levels.<br><br>**Familial Authority and Birth Mode:** Women living with their spouse during pregnancy had significantly lower anxiety, highlighting how partner presence and family support shape emotional preparedness and decision-making for elective C-sections.<br><br>**Commodification and Medicalization of Labor:** Access to VIP wards, personalized care, and high-quality perioperative nursing—alongside effective provider communication—enhanced psychological readiness and satisfaction, showing how health system factors mediate both emotional experience and perceived control over delivery.

de Loenzien M, Mac QNH, and Dumont A. "Women's Empowerment and Elective Cesarean Section for a Single Pregnancy: A Population-Based and Multivariate Study in Vietnam." BMC Pregnancy Childbirth (England) 21, no. 1 (2021): 3. https://doi.org/10.1186/s12884-020-03482-x.	Women's empowerment and elective cesarean section for a single pregnancy: a population-based and multivariate study in Vietnam.	2021	Vietnam	**Constraint and Empowerment:** Among 1,343 institutional births, women demonstrating higher empowerment—measured by rejection of intimate partner violence (IPV) and mobile phone use—were significantly more likely to request elective cesarean sections (ECS), reflecting increased confidence in negotiating their birth choices.<br><br>**Commodification and Medicalization of Labor:** ECS rates were higher among multiparous women (18.4%) than primiparous women (10.1%), suggesting cumulative influence of prior C-sections and reinforcing medicalized childbirth trajectories.<br><br>**Managing Birth Pain and Fear:** Greater antenatal care engagement combined with empowerment indicators increased ECS uptake, highlighting that informed counseling and access to knowledge can shape elective surgical decisions, particularly among first-time mothers.
Diema Konlan K, Baku EK, Japiong M, Dodam Konlan K, and Amoah RM. "Reasons for Women's Choice of Elective Caesarian Section in Duayaw Nkwanta Hospital." J Pregnancy (United States) 2019 (2019): 2320743. https://doi.org/10.1155/2019/2320743.	Reasons for Women's Choice of Elective Caesarian Section in Duayaw Nkwanta Hospital.	2019	Ghana	**Social Meanings of Cesarean and Vaginal Birth:** 28.2% of women chose elective C-section based on advice from friends or family, who framed vaginal birth as excessively painful, reflecting socially transmitted beliefs about birth experiences.<br><br>**Sexual Function and Vaginal Integrity:** 16.7% of women opted for C-section to preserve sexual function and enable quicker resumption of sexual activity, while 11.5% followed spouse/partner preferences, indicating that personal agency interacts with relational and cultural pressures.<br><br>**Commodification and Medicalization of Labor:** 19.2% chose C-section for religious or ritual reasons (e.g., prophecy or auspicious birth dates), highlighting cultural embedding of medicalized birth; overall, elective C-section prevalence was 35.3% of all births and 42% of all C-sections, substantially above the WHO recommended range of 10–15%.
Doraiswamy S, Billah SM, Karim F, Siraj MS, Buckingham A, and Kingdon C. "Physician-Patient Communication in Decision-Making about Caesarean Sections in Eight District Hospitals in Bangladesh: A Mixed-Method Study." Reprod Health (England) 18, no. 1 (2021): 34. https://doi.org/10.1186/s12978-021-01098-8.	Physician-patient communication in decision-making about Caesarean sections in eight district hospitals in Bangladesh: a mixed-method study.	2021	Bangladesh	**Physician Gender and Medical Paternalism:** In 8 district hospitals, physicians (14/16 interviewed had undergone C-section themselves) dominate decision-making; consent processes rarely involve meaningful patient engagement, with the form serving to protect physician reputation rather than support informed choice.<br><br>**Managing Birth Pain and Fear:** Women's decisions are heavily influenced by prior ultrasounds (all 306 observed women had ≥1 USG; some up to 4), which instilled persistent fear of complications, leading many to accept C-sections as the "safe" option for themselves and their babies.<br><br>**Social Meanings of Cesarean and Vaginal Birth:** Religious beliefs and perceived guilt for attempting home birth contributed to compliance with C-section recommendations, while lack of effective communication and mistrust in public facilities limited women's ability to actively negotiate or refuse intervention.

Douché J and Carryer J. "Caesarean Section in the Absence of Need: A Pathologising Paradox for Public Health?" Nurs Inq (Australia) 18, no. 2 (2011): 143–53. https://doi.org/10.1111/j.1440-1800.2011.00533.x.	Caesarean section in the absence of need: a pathologising paradox for public health?	2011	New Zealand	**Commodification and Medicalization of Labor:** Elective C-section is framed as a way to control bodily change and avoid perceived harm; women in popular media express fear of permanent bodily alteration (e.g., "hips getting too big") and seek C-section early to maintain body image.<br><br>**Social Meanings of Cesarean and Vaginal Birth:** Vaginal birth is culturally constructed as risky or potentially damaging, while elective CS is depicted as modern, chic, and rational ("designer deliveries," "too posh to push"), reinforcing ideals of controlled femininity and socially approved motherhood.<br><br>**Physician Gender and Medical Paternalism**: Professional discourse legitimizes elective CS through terms like "maternal request" and "patient choice," normalizing C-section as a socially sanctioned option, while midwives express concern about lowering thresholds for normal birth and preserving physiological childbirth.
Dweik D, Girasek E, Töreki A, Mészáros G, and Pál A. "Women's Antenatal Preferences for Delivery Route in a Setting with High Cesarean Section Rates and a Medically Dominated Maternity System." Acta Obstet Gynecol Scand (United States) 93, no. 4 (2014): 408–15. https://doi.org/10.1111/aogs.12353.	Women's antenatal preferences for delivery route in a setting with high cesarean section rates and a medically dominated maternity system.	2014	Hungary	**Physician Gender and Medical Paternalism:** In Hungary's medically dominated system, 413 women's antenatal CS preferences were strongly shaped by private obstetricians' influence; women reported that doctors convinced them "CS is the most adequate way," highlighting systemic constraints on autonomous choice.<br><br>**Managing Birth Pain and Fear:** Fears about vaginal birth—specifically potential sexual dysfunction and urinary incontinence—were embedded in beliefs that "CS is more beneficial than VD," driving preference for CS, while trust in "the natural way" and desire for bodily agency reduced CS preference.<br><br>**Care Economy and Convenience:** Women's autonomy was constrained by high system-level CS rates, lack of legal right to elective CS, and minimal midwifery support; previous CS history and attitudinal beliefs were stronger predictors of CS preference than sociodemographics or measured fear.
Enabudoso EJ, Ezeanochie MC, and Olagbuji BN. "Perception and Attitude of Women with Previous Caesarean Section towards Repeat Caesarean Delivery." J Matern Fetal Neonatal Med (England) 24, no. 10 (2011): 1212–14. https://doi.org/10.3109/14767058.2011.565833.	Perception and attitude of women with previous caesarean section towards repeat caesarean delivery.	2011	Nigeria	**Managing Birth Pain and Fear:** Among 139 Nigerian women with prior CS, 44.1% cited fear of pain, 23.5% feared death, and 23.5% feared procedural complications as reasons to refuse repeat cesarean; 38.2% reported concerns that CS represents a "failure of womanhood," highlighting deep gendered anxieties.<br><br>**Familial Authority and Birth Mode:** Spousal influence (8.8%) and socio-cultural stigma significantly shaped decisions, with CS perceived as socially undesirable and reflecting compromised femininity; previous perinatal death increased caution, showing intersecting familial and community pressures.<br><br>**Stratified Access to Surgical Birth:** Provider recommendation influenced acceptance, but cost (23.5%), limited pain management, and quality-of-care deficits constrained uptake, emphasizing the interaction of economic barriers, clinical authority, and systemic shortcomings in elective repeat CS decisions.

Essén B, Binder P, and Johnsdotter S. "An Anthropological Analysis of the Perspectives of Somali Women in the West and Their Obstetric Care Providers on Caesarean Birth." J Psychosom Obstet Gynaecol (England) 32, no. 1 (2011): 10–18. https://doi.org/10.3109/0167482X.2010.547966.	An anthropological analysis of the perspectives of Somali women in the West and their obstetric care providers on caesarean birth.	2011	United Kingdom	**Social Meanings of Cesarean and Vaginal Birth:** Among 39 Somali women in the UK, fear of maternal death was strongly associated with CS (majority linked C-section to mortality), with prior traumatic outcomes (e.g., stillbirth) sometimes motivating eventual acceptance; desire to avoid analgesia and minimize medical intervention shaped reluctance.<br><br>**Familial Authority and Birth Mode:** Community advice often discouraged early hospital arrival, reflecting cultural norms and fear of being labeled as overusing medical services, while retrospective acceptance was influenced by social recognition when the baby survived.<br><br>**Physician Gender and Medical Paternalism:** Providers expressed bias and frustration toward Somali women, citing legal pressures and perceived noncompliance; poor postoperative care and structural constraints amplified fear and limited shared decision-making, contributing to adverse outcomes.
Ezeome IV, Ezugworie JO, and Udealor PC. "Beliefs, Perceptions, and Views of Pregnant Women about Cesarean Section and Reproductive Decision-Making in a Specialist Health Facility in Enugu, Southeast Nigeria." Niger J Clin Pract (India) 21, no. 4 (2018): 423–28. https://doi.org/10.4103/njcp.njcp_413_16.	Beliefs, perceptions, and views of pregnant women about cesarean section and reproductive decision-making in a specialist health facility in Enugu, Southeast Nigeria.	2018	Nigeria	**Familial Authority and Birth Mode:** Male dominance strongly shapes CS decisions—82% of women would accept CS only with husband's consent, and 90% believed the husband should sign consent—indicating women's reproductive autonomy is highly constrained by family hierarchy.<br><br>**Managing Birth Pain and Fear:** Despite recognizing CS as safe, 67% of women preferred vaginal delivery because it "makes me more of a woman," and cultural/community expectations reinforced this preference; prior traumatic vaginal births motivated some women to opt for CS.<br><br>**Stratified Access to Surgical Birth:** Only 4% of women choosing CS had tertiary education, highlighting how education and awareness intersect with cultural and gendered pressures to mediate access to elective cesarean delivery.
Fenwick J, Staff L, Gamble J, Creedy DK, and Bayes S. "Why Do Women Request Caesarean Section in a Normal, Healthy First Pregnancy?" Midwifery (Scotland) 26, no. 4 (2010): 394–400. https://doi.org/10.1016/j.midw.2008.10.011.	Why do women request caesarean section in a normal, healthy first pregnancy?	2010	Australia	**Managing Birth Pain and Fear:** 100% of participants (n=14) reported being "petrified" of vaginal birth, fearing physical injury or trauma, making fear the primary driver of elective C-section requests.<br><br>**Constraint and Empowerment:** Women sought C-section to maintain control and orchestrate a "panic-free" birth environment; provider acquiescence reinforced this, with doctors validating the procedure as a "responsible choice," embedding medical authority in decision-making.<br><br>**Familial Authority and Birth Mode:** Women's choices were heavily influenced by mothers' traumatic vaginal births and friends' C-sections, illustrating how community and familial narratives shape perceptions of safety and normalize elective C-sections even in low-risk first pregnancies.

Fenwick J, Toohill J, Creedy DK, Smith J, and Gamble J. “Sources, Responses and Moderators of Childbirth Fear in Australian Women: A Qualitative Investigation.” Midwifery (Scotland) 31, no. 1 (2015): 239–46. https://doi.org/10.1016/j.midw.2014.09.003.	Sources, responses and moderators of childbirth fear in Australian women: a qualitative investigation.	2015	Australia	**Managing Birth Pain and Fear:** Among 43 highly fearful women, fear of pain, tearing, and prior traumatic births strongly influenced preference for C-section; many viewed CS as a protective, “controlled” alternative.<br><br>**Physician Gender and Medical Paternalism:** Women reported limited autonomy in decision-making, citing doctors making choices for them (“I’ll just whisk her in and get her done”), illustrating how medical authority and lack of continuity of care reinforce fear-driven elective CS.<br><br>**Care Economy and Convenience:** Presence of supportive family or familiar providers mitigated fear and reduced desire for elective C-section, highlighting the moderating role of social support and continuity in shaping birth preferences.
Gallagher F, Bell L, Waddell G, Benoît A, and Côté N. “Requesting Cesareans without Medical Indications: An Option Being Considered by Young Canadian Women.” Birth (United States) 39, no. 1 (2012): 39–47. https://doi.org/10.1111/j.1523-536X.2011.00511.x.	Requesting cesareans without medical indications: an option being considered by young canadian women.	2012	Canada	**Managing Birth Pain and Fear:** Among 140 young Canadian women, those with favorable attitudes toward cesarean on maternal request (CDMR) reported higher fear of vaginal birth (mean 7.90 vs 5.00) and perceived CS as safer (p=0.002) and less stressful (p=0.001).<br><br>**Familial Authority and Birth Mode:** Peer influence was significant—participants with friends favorable toward CDMR were 7.5 times more likely to favor it themselves (OR 7.5, p=0.013), highlighting social networks as a gendered mechanism shaping delivery preferences.<br><br>**Physician Gender and Medical Paternalism:** Preference for midwifery care was associated with lower likelihood of favoring CDMR (p=0.013), indicating that trusted provider type and perceived support for vaginal birth can moderate elective CS requests.
Ghelichkhani, S., S.Z. Masoumi, K. Oshvandi, F. Kazemi, and M.R. Ebadian. “ATTITUDE OF PREGNANT WOMEN IN CHOOSING THE TYPE OF DELIVERY: A QUALITATIVE STUDY.” Journal of Postgraduate Medical Institute 35, no. 4 (2021): 202–9. https://doi.org/10.54079/jpmi.35.4.2878.	Attitude of pregnant women in choosing the type of delivery: a qualitative study	2021	Iran	**Managing Birth Pain and Fear:** Fear of pain, tearing, and maternal complications strongly influenced cesarean preference; participants cited concerns such as “People are more afraid of stitches and pain” (P-9, 27 yo).<br><br>**Familial Authority and Birth Mode:** Family and spouse experiences shaped decisions—e.g., a husband’s sister’s positive vaginal birth led to his encouragement of vaginal delivery, while maternal negative experiences pushed women toward cesarean.<br><br>**Physician Gender and Medical Paternalism:** Health system factors, including staff behavior, lack of privacy, and equipment concerns, influenced delivery choice: “The staff…do not like to hear the shouts…I don’t want to have a delivery in front of others.” Respectful care and infrastructure improvements were seen as critical to supporting vaginal delivery.

Ghotbi F, Akbari Sene A, Azargashb E, et al. "Women's Knowledge and Attitude towards Mode of Delivery and Frequency of Cesarean Section on Mother's Request in Six Public and Private Hospitals in Tehran, Iran, 2012." J Obstet Gynaecol Res (Australia) 40, no. 5 (2014): 1257–66. https://doi.org/10.1111/jog.12335.	Women's knowledge and attitude towards mode of delivery and frequency of cesarean section on mother's request in six public and private hospitals in Tehran, Iran, 2012.	2014	Iran	**Managing Birth Pain and Fear:** Fear of labor pain was the main reason for 35.5% of mothers requesting cesarean delivery on maternal request (CDMR).<br><br>**Stratified Access to Surgical Birth:** CDMR was higher among university-educated women (76.3% vs lower education), working women (76.6% vs 47% housewives), insured mothers (57% vs 28.6% uninsured), and those in private hospitals, highlighting socioeconomic and institutional influences.
Gwacham-Anisiobi U, Oladimeji A, Yesufu V, Kurinczuk JJ, Nair M, and McLeish J. "'Is She Pregnant with Jesus?' Exploring Sociocultural Obstacles to Following Medical Advice in the Context of Stillbirth Prevention in Nigeria." BMC Pregnancy Childbirth (England) 25, no. 1 (2025): 593. https://doi.org/10.1186/s12884-025-07646-5.	"Is she pregnant with Jesus?" exploring sociocultural obstacles to following medical advice in the context of stillbirth prevention in Nigeria.	2025	Nigeria	**Social Meanings of Cesarean and Vaginal Birth:** Sociocultural norms in Imo State equate short, non-interventional vaginal births with ideal womanhood; women face stigma, criticism from community, and pressure from family to conform, leading some to delay or avoid medically indicated interventions like cesarean sections.<br><br>**Constraint and Empowerment:** Women's autonomy is limited by family, spouse, and community power dynamics; religious beliefs (e.g., Pentecostal teachings about faith and natural birth) and reliance on traditional birth attendants further reduce compliance with medical advice, sometimes increasing stillbirth risk.<br><br>**Medico-Legal Environment and Defensive Obstetrics:** Lack of trust in healthcare providers, combined with fear of social judgement, delays consent and engagement with hospital services; multilevel, culturally tailored interventions including peer counseling, male/family inclusion, and faith-integrated health messaging are recommended to improve maternal health adherence.
Haines H, Rubertsson C, Pallant JF, and Hildingsson I. "Womens' Attitudes and Beliefs of Childbirth and Association with Birth Preference: A Comparison of a Swedish and an Australian Sample in Mid-Pregnancy." Midwifery (Scotland) 28, no. 6 (2012): e850-6. https://doi.org/10.1016/j.midw.2011.09.011.	Womens' attitudes and beliefs of childbirth and association with birth preference: a comparison of a Swedish and an Australian sample in mid-pregnancy.	2012	Sweden, Australia	**Commodification and Medicalization of Labor:** Women preferring C-section were significantly less likely to endorse the "Birth as a Natural Event" subscale (Sweden $p<0.001$; Australia $p<0.001$), reflecting the influence of medicalized birth cultures and perceptions of birth as risky or needing intervention.<br><br>**Stratified Access to Surgical Birth:** Previous C-section strongly predicted preference for repeat C-section, often shaped by the advice and framing of the medical team; in high-C-section prevalence contexts (Australia), women were more ambivalent and accepting of surgical intervention.<br><br>**Physician Gender and Medical Paternalism:** Midwife-led care in Sweden, emphasizing autonomy and personal control, was associated with stronger preference for vaginal birth, suggesting that system-level support for maternal agency can reduce elective C-section requests.

Hajian, S., M. Shariati, K.M. Najmabadi, M. Yunesian, and M.E. Ajami. "Psychological Predictors of Intention to Deliver Vaginally through the Extended Parallel Process Model: A Mixed-Method Approach in Pregnant Iranian Women." Oman Medical Journal 28, no. 6 (2013): 395–403. https://doi.org/10.5001/omj.2013.115.	Psychological predictors of intention to deliver vaginally through the extended parallel process model: A mixed-method approach in pregnant Iranian women	2013	Iran	**Managing Birth Pain and Fear:** Fear of labor pain and low self-efficacy strongly predicted first-time mothers' intention to avoid vaginal birth; quantitative analysis showed self-efficacy was a significant positive predictor of planning vaginal delivery.<br><br>**Familial Authority and Birth Mode:** Recommendations from mothers, mothers-in-law, spouses, and obstetricians heavily shaped women's intentions, with many deferring to family or physician guidance ("I believe in my doctor's decision…").<br><br>**Social Meanings of Cesarean and Vaginal Birth:** Cultural beliefs and misconceptions about childbirth, including perceived risks of vaginal birth, intersected with limited birth support (e.g., spouse not present), reinforcing reliance on medicalized birth and limiting maternal agency.
Hildingsson I. "Swedish Couples' Attitudes towards Birth, Childbirth Fear and Birth Preferences and Relation to Mode of Birth - a Longitudinal Cohort Study." Sex Reprod Healthc (Netherlands) 5, no. 2 (2014): 75–80. https://doi.org/10.1016/j.srhc.2014.02.002.	Swedish couples' attitudes towards birth, childbirth fear and birth preferences and relation to mode of birth - a longitudinal cohort study.	2014	Sweden	**Managing Birth Pain and Fear:** Women with high childbirth fear prioritized avoiding pain, minimizing stress, controlling timing, and planning birth; fear was strongly associated with preference for planned CS.<br><br>**Social Meanings of Cesarean and Vaginal Birth:** Women who viewed birth as "not natural" and prioritized recovery and breastfeeding scored higher for CS preference; ~7.7% overall preferred elective CS, showing cultural valuation of natural birth moderates uptake.<br><br>**Physician Gender and Medical Paternalism:** Swedish obstetricians retain decision authority over CS, guided by national recommendations; men's attitudes emphasizing doctor decision-making or lack of fear for birth were associated with higher vaginal birth rates (77.7% when neither parent feared or preferred CS).
Högberg, U., N. Lynöe, and M. Wulff. "Cesarean by Choice? Empirical Study of Public Attitudes." Acta Obstetricia et Gynecologica Scandinavica 87, no. 12 (2008): 1301–8. https://doi.org/10.1080/00016340802482978.	Cesarean by choice? Empirical study of public attitudes	2008	Sweden	**Social Meanings of Cesarean and Vaginal Birth:** One-third of respondents believed women should autonomously decide mode of delivery, citing bodily integrity and self-determination, while two-thirds required medical justification, highlighting societal norms limiting elective CS acceptance.<br><br>**Physician Gender and Medical Paternalism:** Public concerns included system strain, increased costs, and perceived medical risks for mother and baby; attitudes shifted toward opposing maternal-request CS when safety evidence was presented, showing deference to medical authority.<br><br>**Commodification and Medicalization of Labor:** Age, urban/rural residence, reproductive history, and trust in healthcare strongly influenced attitudes; low trust correlated with feelings of insecurity, prior dismissal, and perceived maltreatment, revealing structural and gendered dimensions of obstetric decision-making.

Hopkins K. “Are Brazilian Women Really Choosing to Deliver by Cesarean?” Soc Sci Med (England) 51, no. 5 (2000): 725–40. https://doi.org/10.1016/s0277-9536(99)00480-3.	Are Brazilian women really choosing to deliver by cesarean?	2000	Brazil	**Physician Gender and Medical Paternalism:** Doctors actively construct demand for cesarean sections, using professional authority to influence women’s “choices,” with convenience, scheduling, and financial incentives (private hospitals pay ~2× more for C-sections) driving high rates—approaching 90% in private settings.<br><br>**Sexual Function and Vaginal Integrity:** Women report C-sections are perceived to aid faster postpartum recovery and preserve body/sexual function, but first-time mothers largely prefer vaginal birth, showing tension between embodied preferences and socially/normatively constructed beliefs.<br><br>**Care Economy and Convenience:** Public hospitals rarely honor maternal requests for C-sections without strong medical indications, highlighting structural inequities; physician-driven incentives disproportionately shape cesarean rates in private care, reinforcing gendered power asymmetries over women’s reproductive autonomy.
Huang SY, Sheu SJ, Tai CJ, Chiang CP, and Chien LY. “Decision-Making Process for Choosing an Elective Cesarean Delivery among Primiparas in Taiwan.” Matern Child Health J (United States) 17, no. 5 (2013): 842–51. https://doi.org/10.1007/s10995-012-1062-3.	Decision-making process for choosing an elective cesarean delivery among primiparas in Taiwan.	2013	Taiwan	**Managing Birth Pain and Fear:** Women chose elective cesarean delivery (ELCD) primarily to control childbirth risks, avoid pain, and reduce uncertainty, progressing through a three-phase decision process: pre-decision (negative perception of vaginal birth), in-decision (risk assessment across safety, comfort, health, efficiency, aesthetics, timing, and finances), and post-decision (firm commitment and persuasion of family/providers).<br><br>**Sexual Function and Vaginal Integrity:** Decisions were strongly influenced by gendered concerns about vaginal changes, sexual attractiveness, and “feminine charms,” with fears of vaginal loosening, aesthetic body changes, and loss of sexual quality shaping preference for ELCD.<br><br>**Care Economy and Convenience:** Scheduling convenience for spouse, insurance incentives, and financial considerations facilitated ELCD uptake, while provider involvement and early counseling were critical, as decisions were difficult to alter once women reached the post-decision phase.
Hyman, David A., Sarina Taheri, and Mohammad H. Rahmati. “The Impact of Substantial Financial Incentives on C-Section Rates: Evidence from Iran.” Advances in Global Health 3, no. 1 (2024): 2317379. https://doi.org/10.1525/agh.2024.2317379.	The impact of substantial financial incentives on C-section rates: Evidence from Iran.	2024	Iran	**Care Economy and Convenience:** Misaligned financial structures in nonpublic hospitals (patients pay 25% more for vaginal delivery than for C-section) created perverse incentives, contributing to higher C-section rates despite policy efforts.<br><br>**Physician Gender and Medical Paternalism:** Economic incentives for physicians (higher pay for vaginal deliveries) accounted for 84% of the observed reduction in C-section rates, showing that physician financial motivations strongly drive practice patterns.<br><br>**Medico-Legal Environment and Defensive Obstetrics:** Patient co-payment effects accounted for the remainder of reductions; overall, structured financial incentives reduced unnecessary C-sections without negatively impacting maternal or neonatal outcomes, highlighting the effectiveness of system-level interventions alongside nonclinical strategies.

Johansson M, Hildingsson I, and Fenwick J. "'As Long as They Are Safe--Birth Mode Does Not Matter' Swedish Fathers' Experiences of Decision-Making around Caesarean Section." Women Birth (Netherlands) 27, no. 3 (2014): 208–13. https://doi.org/10.1016/j.wombi.2014.03.003.	'As long as they are safe--birth mode does not matter' Swedish fathers' experiences of decision-making around caesarean section.	2014	Sweden	**Managing Birth Pain and Fear:** Swedish fathers prioritized the safety of the mother and baby over birth mode; 100% were supportive of cesarean when recommended, with some expressing personal preference due to fear or concern for partner well-being.<br><br>**Physician Gender and Medical Paternalism:** Fathers largely deferred to obstetricians' authority, equating involvement with being "present" or receiving information; medical professionals' framing of birth as high-risk shifted decision-making power away from couples.<br><br>**Familial Authority and Birth Mode:** Fathers demonstrated limited understanding of cesarean complications, reflecting influence of normalized surgical birth discourses; engagement of both partners in evidence-based decision-making is needed to support informed consent.
Kamal P, Dixon-Woods M, Kurinczuk JJ, Oppenheimer C, Squire P, and Waugh J. "Factors Influencing Repeat Caesarean Section: Qualitative Exploratory Study of Obstetricians' and Midwives' Accounts." BJOG (England) 112, no. 8 (2005): 1054–60. https://doi.org/10.1111/j.1471-0528.2005.00647.x.	Factors influencing repeat caesarean section: qualitative exploratory study of obstetricians' and midwives' accounts.	2005	United Kingdom	**Physician Gender and Medical Paternalism:** Obstetricians and midwives often override women's preferences for repeat C-section, citing perceived psychological fragility or prior trauma; paternalistic norms justify limiting agency even when VBAC is clinically safe.<br><br>**Medico-Legal Environment and Defensive Obstetrics:** Providers shift responsibility to women to avoid litigation and default to surgical delivery when counseling time is limited, reflecting systemic pressures, risk management, and time constraints.<br><br>**Managing Birth Pain and Fear:** Women's fear, misinformation from peers, and lack of support are exploited by providers' subtle persuasion toward C-section, while national VBAC guidelines are referenced inconsistently, highlighting tension between clinical evidence, gendered norms, and professional discretion.
Kennedy HP, Grant J, Walton C, and Sandall J. "Elective Caesarean Delivery: A Mixed Method Qualitative Investigation." Midwifery (Scotland) 29, no. 12 (2013): e138-44. https://doi.org/10.1016/j.midw.2012.12.008.	Elective caesarean delivery: a mixed method qualitative investigation.	2013	England	**Managing Birth Pain and Fear:** Women with prior traumatic or difficult births often opted for elective cesarean delivery (ECD) to avoid repeat trauma, weighing fear of labor heavily in decision-making.<br><br>**Physician Gender and Medical Paternalism:** Clinician bias toward vaginal birth and VBAC as the institutional default influenced how women's choices were framed; counselling in VBAC clinics often steered decisions in line with guidelines rather than individual preference.<br><br>**Care Economy and Convenience:** Access to ECD or preferred birth settings was limited by staff availability, scheduling, and institutional policies (e.g., ECD before 39 weeks restricted), showing that structural and cultural factors shape women's perceived autonomy and ability to exercise choice.

Kolip P. "Attitudes to Cesarean Delivery: The View of Cesarean Section Mothers." Gesundheitswesen (Germany) 70, nos. 8–9 (2008): e22-8. https://doi.org/10.1055/s-2008-1081206.	Attitudes to cesarean delivery: the view of cesarean section mothers.	2008	Germany	**Social Meanings of Cesarean and Vaginal Birth:** Women are ambivalent about cesarean delivery—while 68.9% believe it should be reserved for emergencies and 86.6% think vaginal birth should be attempted first, planned cesareans are perceived as safer, simpler, and less stressful for the child.<br><br>**Commodification and Medicalization of Labor:** Lifestyle factors and celebrity influence affect attitudes—75.2% of women reported that cesarean rates increase in groups where celebrities have cesareans—highlighting social normalization of surgical birth.<br><br>**Familial Authority and Birth Mode:** Women emphasize that "a woman should be able to decide for herself" about cesarean delivery, but only 18.2% agreed that labor pain should be avoided via cesarean, reflecting tension between personal empowerment, traditional views of childbirth, and perceived medical necessity.
Kornelsen, J., E. Hutton, and S. Munro. "Influences on Decision Making Among Primiparous Women Choosing Elective Caesarean Section in the Absence of Medical Indications: Findings From a Qualitative Investigation." Journal of Obstetrics and Gynaecology Canada 32, no. 10 (2010): 962–69. https://doi.org/10.1016/S1701-2163(16)34684-9.	Influences on Decision Making Among Primiparous Women Choosing Elective Caesarean Section in the Absence of Medical Indications: Findings From a Qualitative Investigation	2010	Canada	**Managing Birth Pain and Fear:** Fear of labor pain and negative outcomes, often shaped by personal, family, and peer experiences, was a major motivator for choosing elective cesarean; advanced maternal age and family history of C-section further heightened perceived risk.<br><br>**Constraint and Empowerment:** Women emphasized a strong sense of reproductive rights and autonomy, perceiving the ability to choose delivery mode as inviolable, yet social judgment from peers and family influenced their decisions.<br><br>**Physician Gender and Medical Paternalism:** Obstetrician support facilitated access to elective C-section, while reluctance from family physicians or restrictive hospital policies acted as barriers, demonstrating how provider attitudes directly shape patient choice and access.
Kpanake, L., C. Mullet-Muñoz, M. Pissa, and V. Adjiwanou. "Why Are Pregnant Women in Togo Reluctant to Undergo Caesarean Section? A Systematic Inventory of Motives." Journal of Psychosomatic Obstetrics and Gynecology 45, no. 1 (2024). https://doi.org/10.1080/0167482X.2024.2423628.	Why are pregnant women in Togo reluctant to undergo caesarean section? A systematic inventory of motives	2024	Togo	**Managing Birth Pain and Fear:** 68% of women reported fear of death during C-section due to limited surgical safety in SSA, while cultural norms valorizing vaginal birth stigmatized cesarean as a sign of weakness or failure.<br><br>**Familial Authority and Birth Mode:** Husbands and family exert strong decision-making power; 45% of women feared spouse disapproval and perceived C-section as threatening fertility or family cohesion, limiting women's autonomy.<br><br>**Care Economy and Convenience:** High direct and indirect costs of surgery—including partial government coverage—discouraged uptake, particularly among women in informal labor, while long recovery interfered with household and economic roles.

Lataifeh I, Zayed F, Al-Kuran O, Al-Mehaisen L, Khriesat W, and Khader Y. "Jordanian Obstetricians' Personal Preference Regarding Mode of Delivery." Acta Obstet Gynecol Scand (United States) 88, no. 6 (2009): 733–36. https://doi.org/10.1080/00016340902842444.	Jordanian obstetricians' personal preference regarding mode of delivery.	2009	Jordan	**Sexual Function and Vaginal Integrity:** 86.4% of obstetricians cited fear of long-term perineal damage (stress incontinence, anal sphincter injury) and 50% cited sexual function preservation as reasons for preferring elective C-section for themselves or their wives.<br><br>**Constraint and Empowerment:** Despite personal fears, the majority of obstetricians still preferred vaginal delivery in uncomplicated pregnancies, reflecting professional knowledge and cultural norms that frame CS as unnecessary.<br><br>**Physician Gender and Medical Paternalism:** Younger obstetricians and female obstetricians were slightly more likely to prefer elective CS (14.1% vs 3.7%), indicating intersections of age, gender, and perception of childbirth risk in shaping provider preferences.
Lawani, L.O., F.N. Igboke, C.I. Ukaegbe, et al. "Perception and Socio-Cultural Barriers to the Acceptance of Caesarean Delivery in a Tertiary Hospital in Abakaliki, South East Nigeria." International Journal of Women's Health and Reproduction Sciences 7, no. 2 (2019): 163–68. https://doi.org/10.15296/ijwhr.2019.27.	Perception and socio-cultural barriers to the acceptance of caesarean delivery in a tertiary hospital in Abakaliki, South East Nigeria	2019	Nigeria	**Managing Birth Pain and Fear:** 29.2% of women who refused a previous C-section cited fear of being perceived as weak or as reproductive failure; 8.3–21.4% cited fear of death, reflecting that socio-cultural stigma and mortality concerns strongly influence refusal.<br><br>**Familial Authority Over Birth Mode:** Desire for large family size (4.2% previously) and religious beliefs (12.5–12.9%) constrained women's willingness to accept CS, demonstrating that household, spiritual, and societal expectations shape decision-making.<br><br>**Stratified Access to Surgical Birth:** High out-of-pocket costs (10–20.8%) intersected with cultural barriers, showing that economic constraints compound socio-cultural reluctance, despite >80% awareness of CS indications among antenatal attendees.
Lawrie FA, Mitchell YA, Barrett-Young A, and Clifford AE. "Birth by Emergency Caesarean Delivery: Perspectives of Wāhine Māori in Aotearoa New Zealand." J Health Psychol (England) 29, no. 12 (2024): 1307–20. https://doi.org/10.1177/13591053231218667.	Birth by emergency caesarean delivery: Perspectives of Wāhine Māori in Aotearoa New Zealand.	2024	New Zealand	**Commodification and Medicalization of Labor:** 70% of Wāhine Māori reported feeling treated as "a number" with limited autonomy and rushed care; health system prioritized clinical efficiency over individualized needs, reducing capacity to voice preferences.<br><br>**Managing Birth Pain and Fear:** 100% of participants described emergency C-section experiences as psychologically traumatic, with profound emotional impact including sadness, crying, and need for therapy ("I needed so much therapy to digest [my C-section]").<br><br>**Social Meanings of Cesarean and Vaginal Birth:** Lack of ability to perform Māori birthing rituals undermined cultural wellbeing; culturally affirming care, honoring Māori traditions and values, was essential to positive birth experiences, highlighting the intersection of gender with Indigeneity and the ongoing effects of colonialism and marginalization.

Li WY, Liabsuetrakul T, and Stray-Pedersen B. “Change of Childbirth Preference after Delivery among Nulliparous Chinese Women and Their Partners.” J Obstet Gynaecol Res (Australia) 40, no. 1 (2014): 184–91. https://doi.org/10.1111/jog.12153.	Change of childbirth preference after delivery among nulliparous Chinese women and their partners.	2014	China	**Commodification and Medicalization of Labor:** Post-partum, 25–30% of nulliparous women changed their preference toward cesarean, primarily due to fear of labor pain, perceived safety for mother/baby, and inadequate support or pain management during vaginal birth.<br><br>**Familial Authority and Birth Mode:** Partner preference strongly influenced women’s choices; women whose partners preferred cesarean were 5.5 times more likely to switch to cesarean (adjusted OR = 5.5, 95% CI 1.1–28.0, P = 0.03).<br><br>**Care Economy and Convenience:** Among women maintaining vaginal delivery preference, 90.6% cited faster recovery and 46.8% the natural process; minor concerns included sexual satisfaction (3.3%) and body image (1.7%), highlighting that both personal values and systemic gaps (pain management, routine episiotomy) shape delivery decisions.
Liamputtong P and Watson LF. “The Meanings and Experiences of Cesarean Birth among Cambodian, Lao and Vietnamese Immigrant Women in Australia.” Women Health (United States) 43, no. 3 (2006): 63–82. https://doi.org/10.1300/J013v43n03_04.	The meanings and experiences of cesarean birth among Cambodian, Lao and Vietnamese immigrant women in Australia.	2006	Australia	**Commodification and Medicalization of Labor:** C-section was perceived as “normal” and increasingly routine in Australia; some women viewed it as high-status, offering social prestige and avoidance of vaginal stretching, reflecting both medicalization and cultural interpretations of birth (67 women studied).<br><br>**Physician Gender and Medical Paternalism:** Limited English proficiency strongly influenced mode of delivery—women with poor English were twice as likely to undergo C-section; trust in medical authority and perceived pressure from providers shaped decisions, with inadequate interpreter support amplifying power imbalances.<br><br>**Constraint and Empowerment:** C-section conflicted with traditional postpartum practices (e.g., “mother roasting”) and challenged women’s cultural ideology of motherhood, highlighting intersections of migrant status, gender, and cultural alienation in shaping birth experiences.
Litorp H, Mgaya A, Kidanto HL, Johnsdotter S, and Essén B. “‘What about the Mother?’ Women’s and Caregivers’ Perspectives on Caesarean Birth in a Low-Resource Setting with Rising Caesarean Section Rates.” Midwifery (Scotland) 31, no. 7 (2015): 713–20. https://doi.org/10.1016/j.midw.2015.03.008.	'What about the mother?' Women's and caregivers' perspectives on caesarean birth in a low-resource setting with rising caesarean section rates.	2015	Tanzania	**Managing Birth Pain and Fear:** All women reported limited decision-making power over C-section; acceptance was primarily driven by fear of fetal loss (“I had to be brave so that my baby would come out safe”), reflecting caregiver-dominated decisions and trust in medical authority over personal autonomy.<br><br>**Care Economy and Convenience:** Community narratives of vaginal birth danger and circulating horror stories (paralysis, retained instruments, severe pain, infection) reinforced perception of C-section as both safer for the baby and socially stigmatized, creating psychological and social pressure to accept CS.<br><br>**Constraint and Empowerment:** Women reported post-C-section economic dependency, restricted ability to work, and concerns about fertility and family size, highlighting the intersection of clinical decision-making with social, economic, and gendered constraints in low-resource settings.

Litorp, H., A. Mgaya, C.K. Mbekenga, H.L. Kidanto, S. Johnsdotter, and B. Essén. “Fear, Blame and Transparency: Obstetric Caregivers’ Rationales for High Caesarean Section Rates in a Low-Resource Setting.” Social Science and Medicine 143, no. (Litorp H., helena.litorp@kbh.uu.se; Mgaya A., andrew.mgaya@kbh.uu.se; Mbekenga C.K., columba@muhas.ac.tz; Kidanto H.L., hkidanto@yahoo.co.uk; Essén B., birgitta.essen@kbh.uu.se) International Maternal and Child Health (IMCH), Department of Women’s and Ch (2015): 232–40. https://doi.org/10.1016/j.socscimed.2015.09.003	Fear, blame and transparency: Obstetric caregivers' rationales for high caesarean section rates in a low-resource setting	2015	Tanzania	**Managing Birth Pain and Fear:** Maternal requests for cesarean were reported but often overestimated; caregivers perceived women as unable to tolerate labor pain, constraining women’s agency within a system dominated by provider assumptions.<br><br>**Care Economy and Convenience:** Private practice incentives (“With CS I minimize my time and I earn more!”) and hierarchical team structures drove defensive or convenience-based CS, with fear of blame and audit transparency amplifying overuse.<br><br>**Stratified Access to Surgical Birth:** Shortages of staff and essential equipment (e.g., ultrasound, cardiotocographs) limited safe monitoring of labor, prompting precautionary CS; systemic pressures and poor team communication were key determinants of high CS rates beyond clinical necessity.
Liu TC, Chen CS, Tsai YW, and Lin HC. “Taiwan’s High Rate of Cesarean Births: Impacts of National Health Insurance and Fetal Gender Preference.” Birth (United States) 34, no. 2 (2007): 115–22. https://doi.org/10.1111/j.1523-536X.2007.00157.x.	Taiwan's high rate of cesarean births: impacts of national health insurance and fetal gender preference.	2007	Taiwan	**Social Meanings of Cesarean and Vaginal Birth:** Male fetuses were more likely to be delivered by cesarean than female fetuses due to parental preference for boys and cultural superstitions around auspicious birth dates (e.g., “lucky days” or Year of the Dragon), reflecting gendered fetal preference rather than maternal autonomy.<br><br>**Commodification and Medicalization of Labor:** Taiwan’s National Health Insurance did not directly incentivize cesarean delivery; financial drivers were not a major contributor to high CS rates, indicating systemic insurance structures had minimal impact on mode of delivery.
Liu TC, Lin HC, Chen CS, and Lee HC. “Obstetrician Gender and the Likelihood of Performing a Maternal Request for a Cesarean Delivery.” Eur J Obstet Gynecol Reprod Biol (Ireland) 136, no. 1 (2008): 46–52. https://doi.org/10.1016/j.ejogrb.2007.02.007.	Obstetrician gender and the likelihood of performing a maternal request for a cesarean delivery.	2008	Taiwan	**Physician Gender and Medical Paternalism:** Male obstetricians were more likely to perform elective cesarean upon maternal request in lower-level facilities—district hospitals (OR = 1.53) and OB/GYN clinics (OR = 2.26)—while teaching hospitals mitigated this gender effect due to institutional rules.<br><br>**Constraint and Empowerment:** Maternal age was positively associated with requesting elective CS across all healthcare settings, indicating that older women’s preferences may be more likely to influence delivery mode, but agency remains shaped by provider behavior and institutional context.<br><br>**Commodification and Medicalization of Labor:** Financial incentives (ability to charge extra for CS in clinics) and differences in physician communication—female obstetricians engaging in partnership-building versus male obstetricians being more verbally dominant—may contribute to the higher likelihood of CS by male providers in lower-level institutions.

Liu NH, Mazzoni A, Zamberlin N, et al. 2013. "Preferences for Mode of Delivery in Nulliparous Argentinean Women: A Qualitative Study." Reprod Health (England) 10 (1): 2. https://doi.org/10.1186/1742-4755-10-2.	Preferences for mode of delivery in nulliparous Argentinean women: a qualitative study.	2013	Argentina	**Constraint and Empowerment:** Women generally preferred vaginal delivery, viewing it as natural and a rite of passage, with labor pain framed positively; cesarean was perceived primarily as medically necessary for complications, with fears about slow recovery, greater pain, loss of autonomy, and the belief that future births would also require CS.<br><br>**Social Meanings of Cesarean and Vaginal Birth:** Decision-making varied by sector: non-public sector women exercised greater agency in delivery mode, whereas public sector women were more passive, highlighting systemic inequalities in autonomy; preferences were shaped by provider relationships, cultural norms, and social expectations.<br><br>**Care Economy and Convenience:** Predictability and perceived control over timing motivated consideration of CS for some women, but trust in medical guidance and provider influence often framed CS as a clinical, not elective, choice, reflecting provider-centered decision-making.
Maitanmi BT, Oluyomi OV, Aderemi IO, et al. "Knowledge, Attitude and Perception of Cesarean Section among Pregnant Women Attending Antenatal Clinic at Babcock University Teaching Hospital, Ilishan-Remo, Ogun State." J Matern Fetal Neonatal Med (England) 36, no. 2 (2023): 2278019. https://doi.org/10.1080/14767058.2023.2278019 .	Knowledge, attitude and perception of cesarean section among pregnant women attending antenatal clinic at Babcock University Teaching Hospital, Ilishan-Remo, Ogun State.	2023	Nigeria	**Social Meanings of Cesarean and Vaginal Birth:** Around one-third of women had poor perception of CS, with 20% unwilling to undergo it even if medically indicated; misconceptions included beliefs that CS is for "weak women," reduces dignity, or limits number of children.<br><br>**Care Economy and Convenience:** Overall knowledge and attitude toward CS were good, but targeted antenatal education and supportive interventions (e.g., mHealth tools) are recommended to address gaps, combat cultural misconceptions, and improve informed decision-making.
Miller, A.C., and T.E. Shriver. "Women's Childbirth Preferences and Practices in the United States." Social Science and Medicine 75, no. 4 (2012): 709–16. https://doi.org/10.1016/j.socscimed.2012.03.051 .	Women's childbirth preferences and practices in the United States	2012	United States	**Care Economy and Convenience:** Hospital birth with physician oversight was considered the safest option by most women (n=135 interviewed; n=56 observed; 127 birth stories analyzed), reflecting reliance on biomedical knowledge and technology to minimize perceived maternal and fetal risk.<br><br>**Physician Gender and Medical Paternalism:** Women's agency in childbirth decision-making was constrained by structural factors, including midwife "deserts," limited provider availability, and economic resources, pushing some toward hospital-based or medically managed births even if their personal or cultural preferences favored natural or home birth.<br><br>**Social Meanings of Cesarean and Vaginal Birth:** Three distinct habitus types—medicalized, natural/religious, and hybrid—shaped women's definitions of safety and risk, influencing C-section utilization; women with strong natural/religious frameworks often viewed interventions, including CS, as spiritually inappropriate, highlighting the interplay of cultural norms with structural and health system factors.

Mitler LK, Rizzo JA, and Horwitz SM. "Physician Gender and Cesarean Sections." J Clin Epidemiol (United States) 53, no. 10 (2000): 1030–35. https://doi.org/10.1016/s0895-4356(00)00221-3.	Physician gender and cesarean sections.	2000	United States	**Physician Gender and Medical Paternalism:** Male physicians performed ~60% of deliveries via C-section (OR 1.38, 95% CI: 1.00–1.88), with the effect strongest in university practice (OR 2.82, 95% CI: 1.43–5.55) and minimal in HMO settings due to restricted physician discretion, showing that non-clinical provider factors influence CS rates under clinical uncertainty.<br><br>**Managing Birth Pain and Fear:** Female physicians may perform fewer C-sections due to better understanding of female physiology and more effective patient communication, suggesting that provider gender can shape patient autonomy and decision-making.<br><br>**Commodification and Medicalization of Labor:** Gender-driven differences in CS decisions highlight a "provider-side" influence, emphasizing the importance of awareness, education, and research on how physician characteristics affect elective and medically uncertain cesarean rates.
Mossialos E, Allin S, Karras K, and Davaki K. "An Investigation of Caesarean Sections in Three Greek Hospitals: The Impact of Financial Incentives and Convenience." Eur J Public Health (England) 15, no. 3 (2005): 288–95. https://doi.org/10.1093/eurpub/cki002.	An investigation of Caesarean sections in three Greek hospitals: the impact of financial incentives and convenience.	2005	Greece	**Commodification and Medicalization of Labor:** Almost half of all deliveries were by cesarean (≈183/376 in public and 193/376 in private hospitals), with women holding private insurance in the private hospital having 7.73 times the odds of CS compared to direct-payment patients, showing strong influence of financial incentives.<br><br>**Care Economy and Convenience:** Physician convenience influenced timing of CS, with cesareans more likely performed on weekdays and during daylight hours, indicating scheduling and workload considerations drive non-medically indicated CS.<br><br>**Constraint and Empowerment:** Housewives were more likely to undergo CS in private hospitals, highlighting how socio-economic and demographic factors intersect with health system incentives to shape maternal access and decision-making.
Munro S, Kornelsen J, and Hutton E. "Decision Making in Patient-Initiated Elective Cesarean Delivery: The Influence of Birth Stories." J Midwifery Womens Health (United States) 54, no. 5 (2009): 373–79. https://doi.org/10.1016/j.jmwh.2008.12.014.	Decision making in patient-initiated elective cesarean delivery: the influence of birth stories.	2009	Canada	**Social Meanings of Cesarean and Vaginal Birth**: Women who requested elective cesarean delivery drew on culturally mediated birth stories, with 17 primiparous participants privileging positive narratives of C-sections over negative or horror stories, consolidating confidence, predictability, and perceived control in their decision-making.<br><br>**Constraint and Empowerment:** Women reported feeling well-informed about risks and benefits and in control of their birth experience, but reliance on selective cultural narratives suggests that patient autonomy is intertwined with socially constructed notions of normalcy, risk, and authority, emphasizing the need for culturally sensitive, individualized counseling by providers.

Murray SF. "Relation between Private Health Insurance and High Rates of Caesarean Section in Chile: Qualitative and Quantitative Study." BMJ (England) 321, no. 7275 (2000): 1501–5. https://doi.org/10.1136/bmj.321.7275.1501.	Relation between private health insurance and high rates of caesarean section in Chile: qualitative and quantitative study.	2000	Chile	**Care Economy and Convenience:** In the private sector, 70% of women had cesarean sections, but only 18% had initially requested one, reflecting provider-driven, non-medical factors such as scheduling efficiency and maximizing use of staff and operating theaters.<br><br>**Physician Gender and Medical Paternalism:** Obstetricians navigated competing demands of private practice—personalized patient care and income generation—by liberal use of CS; two distinct professional attitudes were identified: "vaginalists" advocating for natural birth and "caesareanists/operators" favoring efficiency and elective CS, highlighting provider discretion as a major driver.<br><br>**Stratified Access to Surgical Birth:** Elective CS was framed as safe, convenient, and financially efficient, with some obstetricians agreeing to patient requests to retain clientele; quantitative survey showed 6–32% of privately cared-for women reported wanting CS, indicating a large gap between patient preference and actual high CS rates driven by systemic incentives.
Nabawanuka B, Ngabirano T, and Nankumbi J. "Preference and Determinants of Delivery Mode in Pregnant Women with One Cesarean Scar: A Cross-Sectional Study in Two Urban Ugandan Public Hospitals." BMC Pregnancy Childbirth (England) 25, no. 1 (2025): 127. https://doi.org/10.1186/s12884-025-07263-2.	Preference and determinants of delivery mode in pregnant women with one cesarean scar: a cross-sectional study in two urban Ugandan public hospitals.	2025	Uganda	**Managing Birth Pain and Fear:** 81% of women (95% CI: 74–86%) with one previous cesarean preferred a trial of labor (TOLAC) over repeat cesarean, reflecting strong maternal agency in balancing perceived risks, recovery, and personal values.<br><br>**Social Meanings of Cesarean and Vaginal Birth:** Women concerned about body image and marital reactions to cesarean scars were more likely to choose TOLAC, highlighting how sociocultural expectations about appearance and family influence delivery preferences.<br><br>**Care Economy and Convenience:** Economic considerations affected preferences: employed women or those perceiving CS as affordable were more likely to choose repeat cesarean, whereas financial constraints increased preference for TOLAC; fertility intentions also shaped decisions, with women planning more than four children favoring vaginal birth.
Nisar, N., N.A. Sohoo, and A. Memon. "Knowledge, Attitude and Preferences of Pregnant Women towards Modes of Delivery." Journal of the Liaquat University of Medical and Health Sciences 8, no. 3 (2009): 228–33.	Knowledge, attitude and preferences of pregnant women towards modes of delivery	2009	Pakistan	**Social Meanings of Cesarean and Vaginal Birth:** 83.6% of women (373/466) expressed a positive attitude toward vaginal delivery, citing it as natural and beneficial for mother–baby bonding, despite perceiving labor pain as unpleasant.<br><br>**Physician Gender and Medical Paternalism:** Cesarean section acceptance was primarily driven by doctor's advice, not maternal request; women rarely sought CS themselves, indicating low patient-side agency and emphasizing provider influence in decision-making.

O'Brien C and Newport M. "Prioritizing Women's Choices, Consent, and Bodily Autonomy: From a Continuum of Violence to Women-Centric Reproductive Care." Soc Sci Med (England) 333 (2023): 116110. https://doi.org/10.1016/j.socscimed.2023.116110.	Prioritizing women's choices, consent, and bodily autonomy: From a continuum of violence to women-centric reproductive care.	2013	Mexico	**Commodification and Medicalization of Labor:** Over 50% of births in Oaxaca are C-sections—more than three times the WHO-recommended limit—driven in part by financial incentives, provider convenience, and profit-making schemes. C-sections cost roughly 15,000 pesos vs. 5,000 pesos for vaginal birth, encouraging unnecessary procedures.<br><br>**Physician Gender and Medical Paternalism:** Unnecessary C-sections reflect provider-driven decisions over pregnant persons' autonomy, highlighting systemic obstetric violence and limited informed consent. Structural misogyny and unjust training practices amplify the disempowerment of women.
Oelhafen S, Trachsel M, Monteverde S, Raio L, and Cignacco E. "Informal Coercion during Childbirth: Risk Factors and Prevalence Estimates from a Nationwide Survey of Women in Switzerland." BMC Pregnancy Childbirth (England) 21, no. 1 (2021): 369. https://doi.org/10.1186/s12884-021-03826-1.	Informal coercion during childbirth: risk factors and prevalence estimates from a nationwide survey of women in Switzerland.	2021	Switzerland	**Physician Gender and Medical Paternalism:** ~27% of women reported experiencing informal coercion during childbirth in Switzerland, including 37% experiencing intimidation during emergency C-sections and 7% reporting manipulation. Only ~20% felt they had sufficient time to make decisions, highlighting gendered constraints on autonomy.<br><br>**Medico-Legal Environment and Defensive Obstetrics:** Provider pressures—driven by economic incentives, legal liability concerns, and institutional authority—contributed to interventions, including unnecessary cesarean sections, with coercion more frequent in hospital settings than in birthing centers (risk 3x lower in birthing centers).<br><br>**Managing Birth Pain and Fear:** Women who received childbirth debriefing or clear explanations reported lower rates of coercion, demonstrating that improved communication and informed consent practices can reduce psychological harm, increase satisfaction, and mitigate postpartum depression risk.
Panda S, Daly D, Begley C, et al. "Factors Influencing Decision-Making for Caesarean Section in Sweden - a Qualitative Study." BMC Pregnancy Childbirth (England) 18, no. 1 (2018): 377. https://doi.org/10.1186/s12884-018-2007-7.	Factors influencing decision-making for caesarean section in Sweden - a qualitative study.	2018	Sweden	**Commodification and Medicalization of Labor:** Swedish maternity care emphasizes a "belief in normal birth" as the optimal outcome, with midwife-led continuity of care reducing fear, supporting vaginal delivery, and contributing to low CS rates.<br><br>**Physician Gender and Medical Paternalism:** A collaborative team approach between midwives and obstetricians facilitates shared decision-making, professional consensus, and maintenance of low CS rates, minimizing conflicts and defensive practices.<br><br>**Stratified Access to Surgical Birth:** Lack of privatized labor wards ensures uniform, consistent care across patients, preventing financial or institutional incentives from driving unnecessary CS and supporting equitable access to vaginal birth.

Park YL, Clifton B, Ashraf R, et al. “Patient and Provider Perspectives on Pain and Other Dimensions of Anesthesia Experience for Cesarean Delivery: A Qualitative Study.” Res Sq (United States), ahead of print, 2024. https://doi.org/10.21203/rs.3.rs-4814545/v1.	Patient and provider perspectives on pain and other dimensions of anesthesia experience for cesarean delivery: A qualitative study.	2024	United States	**Managing Birth Pain and Fear:** Women experience anxiety and trauma-related stress during awake cesarean delivery, feeling pressure to endure pain and prioritize the baby’s needs, reflecting gendered expectations of maternal stoicism and sacrifice.<br><br>**Physician Gender and Medical Paternalism:** Lack of respectful communication and dismissal by providers—including judgment based on body weight or birth choices—limits women’s autonomy and negatively affects their perception and acceptance of C-section.<br><br>**Familial Authority and Birth Mode:** Community norms valorizing vaginal birth as the “real” or ideal birth create stigma around cesarean delivery, leading women to attempt to approximate vaginal-birth rituals during C-section to align with social and gendered expectations.
Pomeranz M, Arbib N, Haddif L, Reissner H, Romem Y, and Biron T. “‘In God We Trust’ and Other Factors Influencing Trial of Labor versus Repeat Cesarean Section.” J Matern Fetal Neonatal Med (England) 31, no. 13 (2018): 1777–81. https://doi.org/10.1080/14767058.2017.1326906 .	"In God we trust" and other factors influencing trial of labor versus Repeat cesarean section.	2018	Israel	**Constraint and Empowerment:** Women choosing TOLAC had higher internal and chance-based health locus of control, were more religious, and desired more children (average 4.04 vs 3.01 for ERCD), reflecting greater personal agency in delivery decisions.<br><br>**Physician Gender and Medical Paternalism:** Physician influence (“Powerful Others”) strongly steered women toward ERCD (OR = 2.6), highlighting how provider authority constrains autonomy in delivery choice.<br><br>**Social Meanings / Familial Authority:** Secular women more often opted for ERCD (57%), indicating that religious and community norms shape delivery preferences alongside family planning goals.
Qadeer, R., T.A.U. Razaq, H. Khattak, H. Aman, A. Aman, and S. Akhtar. “Current Cesarean Section Rate and Factors Affecting the Patient Decision Regarding Mode of Delivery.” Journal of Postgraduate Medical Institute 38, no. 4 (2024): 300–304. https://doi.org/10.54079/jpmi.38.4.3489.	Current Cesarean Section Rate and Factors Affecting the Patient Decision Regarding Mode of Delivery	2024	Pakistan	**Managing Birth Pain and Fear:** Fear of vaginal delivery, anxiety over repeated exams, prior negative birth experiences, and perceived risk of complications drove 26–20% of women to request elective C-sections, reflecting limited autonomy and agency in decision-making.<br><br>**Social Meanings of Cesarean and Vaginal Birth:** Social narratives and family influence strongly shaped choices, with 19.9% of women believing vaginal delivery could cause serious complications, and family/social input contributing to decision-making in 26% of cases.<br><br>**Physician Gender and Medical Paternalism:** Provider recommendations encouraged C-sections in ~9.8% of cases without clinical indication, while convenience factors like combining C-section with sterilization influenced 2.9%, showing how healthcare system practices and gender norms interact to increase elective CS rates.

Rajabi A, Maharlouei N, Rezaianzadeh A, et al. “Non-Medical Factors Affecting Antenatal Preferences for Delivery Route and Actual Delivery Mode of Women in Southwestern Iran.” J Matern Fetal Neonatal Med (England) 29, no. 22 (2016): 3622–28. https://doi.org/10.3109/14767058.2016.1140137 .	Non-medical factors affecting antenatal preferences for delivery route and actual delivery mode of women in southwestern Iran.	2016	Iran	**Familial Authority and Birth Mode:** Husband and family preferences strongly influenced decisions, with participants reporting statements like “I chose CS to satisfy my husband/family”.<br><br>**Physician Gender and Medical Paternalism:** Obstetricians’ opinions heavily shaped elective C-section uptake, with items like “My gynecologist believes CS carries fewer complications” showing strong predictive effect (OR for medical influence = 1.150), demonstrating provider authority as a key non-medical driver.<br><br>**Managing Birth Pain and Fear:** Desire for convenience, avoidance of pain, and fear of inadequate maternity care drove choices toward CS (62% of participants, well above WHO recommendations), reflecting limited autonomy and constrained empowerment in decision-making.
Reyes E and Rosenberg K. “Maternal Motives behind Elective Cesarean Sections.” Am J Hum Biol (United States) 31, no. 2 (2019): e23226. https://doi.org/10.1002/ajhb.23226.	Maternal motives behind elective cesarean sections.	2019	United States	**Managing Birth Pain and Fear:** Extreme fear of childbirth strongly drives elective C-section preference; 43.9% of women who preferred C-section reported being “extremely fearful of birth,” highlighting fear as a central psychological motivator.<br><br>**Social Meanings of Cesarean and Vaginal Birth:** Anxiety is amplified by the medicalized U.S. birth culture and negative cultural depictions of labor, which frame vaginal birth as high-risk or undesirable, shaping women’s perceptions and birth preferences.
Ryding EL, Lukasse M, Kristjansdottir H, Steingrimsdottir T, and Schei B. “Pregnant Women’s Preference for Cesarean Section and Subsequent Mode of Birth - a Six-Country Cohort Study.” J Psychosom Obstet Gynaecol (England) 37, no. 3 (2016): 75–83. https://doi.org/10.1080/0167482X.2016.1181055 .	Pregnant women's preference for cesarean section and subsequent mode of birth - a six-country cohort study.	2016	Belgium, Iceland, Denmark, Estonia, Norway, Sweden	**Managing Birth Pain and Fear:** Severe fear of childbirth (FOC), depressive symptoms, and history of childhood/adult abuse strongly predict preference for cesarean section; previous CS with no prior vaginal birth had the strongest association with actual elective CS (OR 17.4).<br><br>**Physician Gender and Medical Paternalism:** Obstetricians often accommodate maternal preference, especially when relative medical indications exist, demonstrating how provider practices and guidelines mediate whether expressed preferences convert into surgery.<br><br>**Social Meanings of Cesarean and Vaginal Birth:** Country-specific norms and deferential attitudes toward clinicians influence CS preference (e.g., Belgian women less likely to express CS preference due to trust in physicians); differences between countries are largely explained by sociodemographic and psychological factors rather than purely cultural context.

Samara, B., and A.R. Sabella. “The Knowledge and Attitudes of Palestinian Women towards Different Childbirth Delivery Options.” Clinical and Experimental Obstetrics and Gynecology 48, no. 1 (2021): 138–43. https://doi.org/10.31083/j.ceog.2021.01.2155.	The knowledge and attitudes of Palestinian women towards different childbirth delivery options	2021	Palestine	**Physician Gender and Medical Paternalism:** Most women believed that the decision to perform a cesarean should rest with doctors rather than themselves, reflecting a strong gendered power imbalance and provider-driven decision-making.<br><br>**Managing Birth Pain and Fear:** Fear of labor pain strongly motivated women’s consideration of cesarean delivery, even though 87% were aware that CS is associated with greater postoperative pain and muscle weakness.<br><br>**Stratified Access to Surgical Birth:** Availability of health insurance increased the tendency toward CS; despite intermediate-to-good knowledge of medical risks (infection 49%, neonatal respiratory disorder 31%, uterine rupture 59%), women’s attitudes were generally unfavorable toward CS, showing limited autonomy in childbirth decisions.
Schantz C, Sim KL, Petit V, Rany H, and Goyet S. “Factors Associated with Caesarean Sections in Phnom Penh, Cambodia.” Reprod Health Matters (England) 24, no. 48 (2016): 111–21. https://doi.org/10.1016/j.rhm.2016.11.009.	Factors associated with caesarean sections in Phnom Penh, Cambodia.	2016	Cambodia	**Social Meanings of Cesarean and Vaginal Birth:** 60% of women requesting C-sections cited protecting genital aesthetics, preserving sexual desirability, or preventing vaginal enlargement as motivations; male partners confirmed preference for “tightness,” showing strong gendered cultural norms influencing delivery choice.<br><br>**Familial Authority and Birth Mode:** Family members, especially mothers-in-law, exerted substantial influence on women’s requests for C-sections; symbolic beliefs about auspicious birth dates, luck, and “energy” reinforced culturally embedded decision-making.<br><br>**Physician Gender and Medical Paternalism:** Providers endorsed C-sections as safer for mother and baby, while higher costs ($278 vs $60 for vaginal birth) and perceptions of C-sections as “for rich and celebrities” shaped access; overuse of ultrasounds contributed to increased elective requests.
Schantz C, Aboubakar M, Traoré AB, Ravit M, de Loenzien M, and Dumont A. “Caesarean Section in Benin and Mali: Increased Recourse to Technology Due to Suffering and under-Resourced Facilities.” Reprod Biomed Soc Online (England) 10 (2020): 10–18. https://doi.org/10.1016/j.rbms.2019.12.001.	Caesarean section in Benin and Mali: increased recourse to technology due to suffering and under-resourced facilities.	2020	Benin, Mali	**Managing Birth Pain and Fear:** Women in Benin and Mali experience intense fear of death, surgery, and pain during childbirth in under-resourced, non-private labor wards, driving both requests for C-sections and heightened maternal distress.<br><br>**Commodification and Medicalization of Labor:** Preventive and provider-driven C-sections arise from systemic resource constraints—limited OR availability, lack of monitoring equipment, and staff burnout—rather than strict medical indication; free C-section policies further increase elective demand.<br><br>**Familial Authority and Birth Mode:** Family opposition, social pressure, and gendered expectations intersect with institutional failures, shaping women’s autonomy and decision-making; women’s preference for vaginal birth is often overridden by systemic and social pressures.

Schantz C, Pantelias AC, de Loenzien M, et al. 2021. "'A Caesarean Section Is like You've Never Delivered a Baby': A Mixed Methods Study of the Experience of Childbirth among French Women." Reprod Biomed Soc Online (England) 12: 69–78. https://doi.org/10.1016/j.rbms.2020.10.003.	'A caesarean section is like you've never delivered a baby': A mixed methods study of the experience of childbirth among French women.	2021	France	**Social Meanings of Cesarean and Vaginal Birth:** 97.5% of women preferred vaginal birth, associating it with "naturalness," authentic motherhood, and maternal identity; C-section was perceived as delegitimizing, disempowering, and emotionally distancing from the baby.<br><br>**Managing Birth Pain and Fear:** Women feared vaginal birth-related bodily damage (pelvic floor, genital changes) yet also viewed C-sections as involving longer recovery and increased pain, highlighting complex risk–benefit perceptions shaped by medicalized childbirth narratives.<br><br>**Physician Gender and Medical Paternalism:** Provider-driven decision-making and limited out-of-hospital birth options reinforced medical authority; caregivers' own preference for vaginal birth shaped interactions and reinforced social norms around "authentic" childbirth.
Schei B, Lukasse M, Ryding EL, et al. "A History of Abuse and Operative Delivery--Results from a European Multi-Country Cohort Study." PLoS One (United States) 9, no. 1 (2014): e87579. https://doi.org/10.1371/journal.pone.0087579.	A history of abuse and operative delivery--results from a European multi-country cohort study.	2014	Belgium, Iceland, Denmark, Estonia, Norway, Sweden	**Managing Birth Pain and Fear:** Adult sexual abuse significantly increased the likelihood of elective cesarean section in nulliparous women (AOR 2.12–4.07), with current suffering from abuse yielding the highest risk (AOR 4.07); psychological distress and fear of vaginal delivery drive avoidance of labor.<br><br>**Social Meanings of Cesarean and Vaginal Birth:** Multiparous women with a history of physical abuse had higher odds of emergency C-section, highlighting how prior trauma constrains women's perceived safety and autonomy in childbirth decisions.<br><br>**Physician Gender and Medical Paternalism:** Providers may respond to trauma-informed concerns by accommodating elective or emergency C-sections, demonstrating the intersection of patient history, psychological safety, and medical decision-making.
Serçekuş P, Egelioglu Cetisli N, and İnci FH. "Birth Preferences by Nulliparous Women and Their Partners in Turkey." Sex Reprod Healthc (Netherlands) 6, no. 3 (2015): 182–85. https://doi.org/10.1016/j.srhc.2015.03.002.	Birth preferences by nulliparous women and their partners in Turkey.	2015	Turkey	**Sexual Function and Vaginal Integrity:** Nulliparous women cited fear of injury to sexual organs, incontinence, and general childbirth pain as key motivators for preferring C-sections.<br><br>**Familial Authority and Birth Mode:** Partners influenced delivery preferences modestly (3.1% of men), while cultural norms around male providers and modesty led some women to prefer C-sections to avoid male doctors attending birth.<br><br>**Social Meanings of Cesarean and Vaginal Birth:** Vaginal birth was favored for naturalness, health benefits, quicker recovery, and earlier breastfeeding; women's perceived maternal instinct and desire for a known doctor also shaped birth choices, highlighting gendered expectations and agency in decision-making.

Shahoei R, Rezaei M, Ranaei F, Khosravy F, and Zaheri F. “Kurdish Women’s Preference for Mode of Birth: A Qualitative Study.” Int J Nurs Pract (Australia) 20, no. 3 (2014): 302–9. https://doi.org/10.1111/ijn.12150.	Kurdish women's preference for mode of birth: a qualitative study.	2014	Iran	**Social Meanings of Cesarean and Vaginal Birth:** Fear of vaginal birth driven by negative stories, perceived risks to mother and baby, and lack of antenatal information, while vaginal birth is valued for naturalness, maternal-infant bonding, and assurance of infant health.<br><br>**Familial Authority and Birth Mode:** Husbands, relatives, and friends strongly shaped preferences—support for vaginal birth encouraged natural delivery, while social pressure and anecdotal C-section narratives pushed some toward surgery.
Shen M and Li L. “Differences in Cesarean Section Rates by Fetal Sex among Chinese Women in the United States: Does Chinese Culture Play a Role?” Econ Hum Biol (Netherlands) 36 (2020): 100824. https://doi.org/10.1016/j.ehb.2019.100824.	Differences in Cesarean section rates by fetal sex among Chinese women in the United States: Does Chinese culture play a role?	2020	United States	**Social Meanings of Cesarean and Vaginal Birth:** Chinese cultural son preference drives higher C-section rates for male infants, particularly for first-borns, reflecting gendered expectations of family lineage and parental investment.<br><br>**Physician Gender and Medical Paternalism:** Differential prenatal behaviors, including higher maternal weight gain and increased macrosomia for boys, create more medical indications for C-sections, illustrating how cultural practices intersect with provider-mediated surgical decisions.<br><br>**Care Economy and Convenience:** Families may schedule C-sections for boys on auspicious dates, showing culturally embedded decision-making; healthcare providers should recognize and counsel around culturally driven demand to reduce non-medically indicated surgeries.
Shi Y, Jiang Y, Zeng Q, et al. “Influencing Factors Associated with the Mode of Birth among Childbearing Women in Hunan Province: A Cross-Sectional Study in China.” BMC Pregnancy Childbirth (England) 16 (2016): 108. https://doi.org/10.1186/s12884-016-0897-9.	Influencing factors associated with the mode of birth among childbearing women in Hunan Province: a cross-sectional study in China.	2016	China	**Physician Gender and Medical Paternalism:** Doctor recommendations strongly influenced elective C-section, with an OR of 24.8 for switching from vaginal birth to C-section; systemic factors such as staff shortages and financial incentives further increased CS rates.<br><br>**Managing Birth Pain and Fear:** 37.3% of women preferring C-section reported lack of confidence in vaginal birth, highlighting fear and psychological barriers as major drivers of elective CS.<br><br>**Familial Authority and Birth Mode:** Husband’s preferred mode strongly affected women’s choices (OR=4.4), reflecting gendered power dynamics and culturally mediated social expectations regarding childbirth.

Stoll K, Fairbrother N, Carty E, et al. "'It's All the Rage These Days': University Students' Attitudes toward Vaginal and Cesarean Birth." Birth (United States) 36, no. 2 (2009): 133–40. https://doi.org/10.1111/j.1523-536X.2009.00310.x.	"It's all the rage these days": University students' attitudes toward vaginal and cesarean birth.	2009	Canada	**Managing Birth Pain and Fear:** Fear of pain and concern about vaginal damage were primary drivers of elective C-section preference among students; ~9% of participants favored C-section, citing pain avoidance and low confidence in vaginal birth.<br><br>**Social Meanings of Cesarean and Vaginal Birth:** Vaginal birth framed as a "natural" and transformative female experience, while cesarean viewed as convenient, fast, and controlled, reflecting cultural narratives about empowerment and bodily integrity.<br><br>**Physician Gender and Medical Paternalism:** Men emphasized health and safety more than personal pain; findings suggest that educational interventions and accurate information about childbirth could shift attitudes and reduce elective C-section preference among young adults.
Sun, Na, Xiaoxv Yin, Lei Qiu, et al. "Factors Associated with Chinese Pregnant Women's Preference for a Cesarean Section Based on the Theory of Planned Behaviour." Tropical Medicine & International Health: TM & IH 25, no. 2 (2020): 209–15. https://doi.org/10.1111/tmi.13323.	Factors associated with Chinese pregnant women's preference for a cesarean section based on the theory of planned behaviour.	2019	China	**Managing Birth Pain and Fear:** Lower childbirth self-efficacy and negative attitudes toward vaginal birth strongly increased the likelihood of preferring a cesarean; nulliparous women planning a second child were less likely to choose CS, highlighting confidence and future reproductive planning as key psychological drivers.<br><br>**Familial Authority and Birth Mode:** Social pressures from family, friends, obstetricians, and media shaped women's CS preferences, indicating that subjective norms and culturally transmitted beliefs (e.g., "once a cesarean, always a cesarean") are significant gendered determinants.<br><br>**Stratified Access to Surgical Birth:** Previous cesarean strongly predicted repeat CS preference, underscoring how prior medical interventions and provider guidance institutionalize surgical birth; interventions targeting provider education, antenatal counseling, and promotion of vaginal birth after cesarean are recommended.
Suwanrath C, Chunuan S, Matemanosak P, and Pinjaroen S. "Why Do Pregnant Women Prefer Cesarean Birth? A Qualitative Study in a Tertiary Care Center in Southern Thailand." BMC Pregnancy Childbirth (England) 21, no. 1 (2021): 23. https://doi.org/10.1186/s12884-020-03525-3.	Why do pregnant women prefer cesarean birth? A qualitative study in a tertiary care center in Southern Thailand.	2021	Thailand	**Managing Birth Pain and Fear:** Fear of labor pain, failed vaginal birth, and harming the baby was a primary driver of elective cesarean preference; prior traumatic birth experiences (e.g., vacuum-assisted deliveries) further amplified fear and desire for surgical birth.<br><br>**Care Economy and Convenience:** Women viewed cesarean as convenient and controllable—allowing scheduled delivery, shorter perceived recovery, and options for concurrent procedures (e.g., tubal ligation), highlighting perceived functional and temporal benefits of surgery.<br><br>**Social Meanings of Cesarean and Vaginal Birth:** Cultural beliefs, including superstitions around auspicious birth dates, influenced CS requests, showing that social and symbolic norms intersect with fear and convenience to shape maternal choice; these beliefs present unique counseling challenges for obstetricians in Thailand.

Tadevosyan M, Ghazaryan A, Harutyunyan A, Petrosyan V, Atherly A, and Hekimian K. “Factors Contributing to Rapidly Increasing Rates of Cesarean Section in Armenia: A Partially Mixed Concurrent Quantitative-Qualitative Equal Status Study.” BMC Pregnancy Childbirth (England) 19, no. 1 (2019): 2. https://doi.org/10.1186/s12884-018-2158-6.	Factors contributing to rapidly increasing rates of cesarean section in Armenia: a partially mixed concurrent quantitative-qualitative equal status study.	2019	Armenia	**Managing Birth Pain and Fear:** Maternal request drove CS, often to avoid labor pain or because of previous difficult vaginal births; women’s insistence sometimes overrode provider attempts to encourage vaginal delivery.<br><br>**Care Economy and Convenience:** Financial incentives strongly influenced provider behavior—bonus payments for CS were 11× higher than for vaginal births, particularly in secondary hospitals, contributing to rapidly rising CS rates.<br><br>**Physician Gender and Medical Paternalism:** Outdated regulations and monitoring gaps created a permissive environment for non-medically indicated CS; women’s autonomy intersected with systemic incentives, enabling elective CS even when not clinically necessary.
Thirukumar P, Henry A, and Coates D. “Women’s Experiences and Involvement in Decision-Making in Relation to Planned Cesarean Birth: An Interview Study.” J Perinat Educ (United States) 30, no. 4 (2021): 213–22. https://doi.org/10.1891/J-PE-D-20-00034.	Women's Experiences and Involvement in Decision-Making in Relation to Planned Cesarean Birth: An Interview Study.	2021	Australia	**Managing Birth Pain and Fear:** Anxiety and previous traumatic births strongly influenced preference for planned cesarean; 8 of 33 women specifically cited these factors as driving their choice.<br><br>**Physician Gender and Medical Paternalism:** Clinician recommendations heavily shaped decisions—many women accepted cesarean if advised as safest for mother or baby, even when preferring vaginal birth; cultural norms (e.g., Southeast Asian backgrounds) reinforced cesarean preference, while social stigma framed vaginal birth as an expected challenge.<br><br>**Medico-Legal Environment and Defensive Obstetrics:** Shared decision-making was inconsistently applied; women desired more involvement, tailored information, longer consultations, and continuity of care, highlighting gaps between clinical guidance and patient autonomy.
Ugwu NU and de Kok B. “Socio-Cultural Factors, Gender Roles and Religious Ideologies Contributing to Caesarian-Section Refusal in Nigeria.” Reprod Health (England) 12 (2015): 70. https://doi.org/10.1186/s12978-015-0050-7.	Socio-cultural factors, gender roles and religious ideologies contributing to Caesarian-section refusal in Nigeria.	2015	Nigeria	**Sexual Function and Vaginal Integrity:** Vaginal birth is culturally framed as a marker of womanhood; women feared cesarean would compromise fertility or social legitimacy, with perceptions of CS as “weak” or “lazy” reported.<br><br>**Constraint and Empowerment:** Decisions about CS were heavily influenced by husbands, in-laws, co-wives, and community pressures; women risked marital consequences or social disapproval if they underwent CS.<br><br>**Stratified Access to Surgical Birth:** Use of alternative providers (TBAs, religious leaders) delayed hospital CS, leading to predominantly emergency interventions; availability of CS in some facilities (e.g., missionary hospitals) increased uptake, but socio-cultural and gendered norms remained the dominant barrier.

Vangen S, Johansen RE, Sundby J, Traeen B, and Stray-Pedersen B. "Qualitative Study of Perinatal Care Experiences among Somali Women and Local Health Care Professionals in Norway." Eur J Obstet Gynecol Reprod Biol (Ireland) 112, no. 1 (2004): 29–35. https://doi.org/10.1016/s0301-2115(03)00313-0.	Qualitative study of perinatal care experiences among Somali women and local health care professionals in Norway.	2004	Norway	**Sexual Function and Vaginal Integrity:** Somali women's fear of labor was heightened by prior circumcision, with vivid trauma-related anxieties; many associated vaginal birth with pain or re-traumatization, while all expressed strong aversion to cesarean birth.<br><br>**Physician Gender and Medical Paternalism:** Lack of provider knowledge and training in defibulation led to some unnecessary cesareans; poor communication and delayed defibulation during labor exacerbated fear and reduced trust.<br><br>**Social Meanings of Cesarean and Vaginal Birth:** Cultural norms and midwives' non-interference attitudes reinforced women's suffering and limited agency; individualized antenatal counseling, discussion of circumcision status, and empowerment strategies are needed to improve outcomes.
Vazquez Corona M, Betrán AP, and Bohren MA. "The Portrayal and Perceptions of Cesarean Section in Mexican Media Facebook Pages: A Mixed-Methods Study." Reprod Health (England) 19, no. 1 (2022): 49. https://doi.org/10.1186/s12978-022-01351-8.	The portrayal and perceptions of cesarean section in Mexican media Facebook pages: a mixed-methods study.	2022	Mexico	**Social Meanings of Cesarean and Vaginal Birth:** Media and public discourse portray C-sections as conflicting with ideals of motherhood and body image, with comments reflecting stigma (e.g., "less of a mother") and fear of scarring or negative health outcomes for the baby (asthma, allergies, obesity).<br><br>**Commodification and Medicalization of Labor:** Private hospitals and media narratives highlight financial incentives and overmedicalization, while systemic sexism and obstetric violence contribute to mistrust and unnecessary C-sections.
Vila Ortiz M, Gialdini C, Hanson C, Betrán AP, Carroli G, and Mølsted Alvesson H. "A Bit of Medical Paternalism? A Qualitative Study on Power Relations between Women and Healthcare Providers When Deciding on Mode of Birth in Five Public Maternity Wards of Argentina." Reprod Health (England) 20, no. 1 (2023): 122. https://doi.org/10.1186/s12978-023-01661-5.	A bit of medical paternalism? A qualitative study on power relations between women and healthcare providers when deciding on mode of birth in five public maternity wards of Argentina.	2023	Argentina	**Physician Gender and Medical Paternalism:** Healthcare providers report tension between respecting women's autonomy under Argentina's Law of Humanized Birth and exercising clinical judgment; women increasingly assert preferences for cesarean sections to avoid labor pain, creating perceived loss of "beneficial power" among providers.<br><br>**Managing Birth Pain and Fear:** Fear of labor pain is a major driver of maternal request for cesarean delivery, with some companions also influencing decisions; high-risk vaginal birth requests are sometimes overridden by providers despite women's insistence.

Walker R, Turnbull D, and Wilkinson C. “Increasing Cesarean Section Rates: Exploring the Role of Culture in an Australian Community.” Birth (United States) 31, no. 2 (2004): 117–24. https://doi.org/10.1111/j.0730-7659.2004.00289.x.	Increasing cesarean section rates: exploring the role of culture in an Australian community.	2004	Australia	**Social Meanings of Cesarean and Vaginal Birth:** Cesarean section is widely perceived in the Australian community as easier, more convenient, and routine, influencing women’s consideration of CS even when medically unnecessary.<br><br>**Managing Birth Pain and Fear:** Women with previous cesarean sections were more likely to consider repeat CS, highlighting the role of prior experiences in shaping risk perception and fear of vaginal birth.
Weeks FH, Sadler M, and Stoll K. “Preference for Caesarean Attitudes toward Birth in a Chilean Sample of Young Adults.” Women Birth (Netherlands) 33, no. 2 (2020): e159–65. https://doi.org/10.1016/j.wombi.2019.03.012.	Preference for caesarean attitudes toward birth in a Chilean sample of young adults.	2020	Chile	**Managing Birth Pain and Fear:** Fear of labor pain was the dominant factor influencing cesarean preference, cited by 70.1% of participants.<br><br>**Commodification and Medicalization of Labor:** Positive attitudes toward technological intervention showed the strongest association with cesarean preference, reflecting the cultural normalization of medicalized birth.<br><br>**Social Meanings of Cesarean and Vaginal Birth:** Misinformation and gaps in knowledge about actual risks of vaginal versus cesarean birth suggest that patient-centered education, including partner involvement, could shift preferences toward vaginal birth.
